# CO and CO_2_ Hydrogenation over CeO_2_ and CeO_2_-Based Composite Catalysts: Defect Chemistry, Interfacial Sites and Selectivity Control

**DOI:** 10.3390/nano16181193

**Published:** 2026-09-21

**Authors:** Guo Tian

**Affiliations:** 1 Key Laboratory of Coal Clean Conversion & Chemical Engineering Process, School of Chemical Engineering, Xinjiang University, Urumqi 830046, China; tg.2020@tsinghua.org.cn; 2 Beijing Key Laboratory of Green Chemical Reaction Engineering and Technology, Department of Chemical Engineering, Tsinghua University, Beijing 100084, China; 3 Ordos Laboratory, Ordos 017010, China

**Keywords:** CeO_2_-based catalysts, CO_2_ hydrogenation, CO hydrogenation, oxygen vacancies, metal–oxide interfaces, selectivity control, in situ catalytic characterization

## Abstract

CO and CO_2_ hydrogenation provide essential routes for linking carbon resource recycling with renewable hydrogen utilization, yet the predictable design of CeO_2_-based catalysts remains constrained by an incomplete understanding of how CeO_2_ defect chemistry couples with interfacial reaction pathways. This review adopts a function-oriented rather than a metal-by-metal enumeration approach, covering pristine CeO_2_, single-metal/CeO_2_, bimetallic/CeO_2_, and CeO_2_–metal oxide composite catalysts. We argue that CeO_2_ should not be viewed merely as an oxygen vacancy reservoir; its catalytic function arises from the coupled effects of exposed crystal facets, Ce^4+^/Ce^3+^ redox cycling, oxygen vacancy formation and migration, surface hydroxyl chemistry, water desorption, and dynamic metal–oxide interfaces. In methanol synthesis, methanation, the reverse water–gas shift reaction, and C_2_ oxygenate formation, the same structural descriptors may promote or suppress the target pathway, depending on oxygen vacancy location, metal nuclearity, oxide basicity, and intermediate binding strength. We further discuss how Cu-Ce interfaces regulate methanol versus CO production, how Ni-, Co-, and Ru-based interfaces promote deep hydrogenation, and how Pd-, Rh-, and composite oxide systems expand oxygenate selectivity. Finally, we emphasize that future research needs to develop in situ descriptors, quantitatively distinguish different types of oxygen vacancies, and evaluate catalyst stability under realistic environments including water-rich, CO-containing, and cyclic operation conditions. Reinterpreting CeO_2_ from a “reducible support” to a “programmable dynamic interface” holds promise for advancing catalyst design for selective CO_x_ hydrogenation to CO, methane, methanol, and higher-carbon oxygenates.

## 1. Introduction

Against the backdrop of the “dual carbon” goals and large-scale grid integration of renewable electricity, CO and CO_2_ hydrogenation are evolving beyond their traditional roles in syngas utilization within the coal-based and natural-gas-based chemical industries. They are now emerging as key routes connecting carbon resource recycling, green hydrogen consumption, and the production of liquid fuels and platform chemicals [1]. Syngas (CO/H_2_) is a highly flexible C_1_ feedstock that can be converted via Fischer–Tropsch synthesis to liquid hydrocarbons, waxes, and olefins, or transformed over Cu/ZnO/Al_2_O_3_-type catalysts into methanol, which can be further upgraded to olefins, aromatics, gasoline, or oxygenated chemicals [2,3,4,5]. Compared with direct combustion of fossil carbon, syngas conversion offers tunable product spectra, mature process fundamentals, and compatibility with diverse carbon sources including coal, biomass, natural gas reforming, industrial off-gases, and CO_2_ reduction.

CO_2_ hydrogenation further endows this reaction network with clear energy-environmental significance [6]. CO_2_ can be converted via the reverse water–gas shift (RWGS) to CO and then enter the conventional syngas conversion network, or it can be directly hydrogenated to methanol, methane, or hydrocarbons, thereby storing otherwise difficult-to-store renewable electricity as chemical energy through green hydrogen [7,8,9,10,11,12]. Among these, CO_2_ methanation can be coupled with Power-to-Gas technologies; methanol synthesis serves as a versatile fuel, solvent, platform molecule, and key carrier in the “methanol economy”; and CO_2_-FT or methanol-mediated routes offer pathways to renewable liquid fuels [7,10,11,12]. However, the CO_2_ molecule is thermodynamically stable with a strong C=O bond, its hydrogenation involves complex pathways, and RWGS, deep methanation, carbon deposition, water-induced deactivation, and thermodynamic equilibrium constraints concurrently affect activity, selectivity, and stability [13].

Conventional syngas conversion catalysts provide important reference points for understanding this balance [14]. In Fischer–Tropsch synthesis, Co and Fe catalysts control hydrocarbon product distribution through CO dissociation, chain growth, and hydrogenation termination, but selectivity is jointly constrained by the Anderson-Schulz-Flory distribution, H_2_/CO ratio, CO_2_/H_2_O, and metal particle structure [2,3]. In industrial methanol synthesis, Cu/ZnO/Al_2_O_3_ relies on Cu-ZnO_x_ interfaces, defective Cu sites, and an appropriate CO_2_ level to maintain activity; Behrens et al. showed that interfacial sites formed between Zn species and defective Cu surfaces are key active structures for methanol generation [4,5]. These classical insights suggest that the key to CO/CO_2_ hydrogenation catalysts is not merely CO_x_ adsorption and activation, but also the coupled control of H_2_ dissociation, oxygenate intermediate stabilization, C-O/C-H bond transformation, water tolerance, and product distribution.

CeO_2_ has attracted considerable attention in CO/CO_2_ hydrogenation because its fluorite lattice can reversibly accommodate Ce^4+^/Ce^3+^ conversion and oxygen-vacancy formation [15,16,17,18,19]. These features provide oxygen storage capacity, tunable redox properties and surface sites capable of polarizing CO_2_ and stabilizing oxygenated intermediates [20,21,22,23]. CeO_2_ should therefore be regarded as a redox-active and defect-regulated oxide rather than an inert support. Another important function of CeO_2_ is the construction of metal–oxide interfaces. At these interfaces, metal sites generally promote H_2_ dissociation and hydrogenation, whereas adjacent CeO_2_ sites contribute to CO_2_ polarization, oxygenate stabilization and water-related processes [24,25]. The catalytic performance therefore depends on the local contact geometry and electronic interaction between the metal and CeO_2_ [26].

A structured literature search was conducted primarily in the Web of Science Core Collection, with Google Scholar used as a supplementary discovery tool. The literature search was conducted in June 2026 and covered publications from 1996 through June 2026. Three concise keyword combinations were used: (1) (“CO_2_ hydrogenation” OR “CO hydrogenation”) AND (CeO_2_ OR ceria); (2) (“CO_2_ methanation” OR “methanol synthesis” OR “reverse water–gas shift”) AND (CeO_2_ OR ceria); and (3) (“oxygen vacancy” OR “metal–support interaction”) AND (CeO_2_ OR ceria) AND (hydrogenation OR CO_2_). The same concepts were adapted to the syntax of each database, and Google Scholar was used to identify additional relevant studies and cited references not retrieved in the primary database searches. Records were screened first by title and abstract, followed by full-text assessment of potentially relevant studies. Duplicate records retrieved from different databases were removed before the final selection. Studies were included when they investigated CeO_2_-containing catalysts for CO or CO_2_ hydrogenation, including methanol synthesis, methanation, reverse water–gas shift, or C^2+^ oxygenate formation, or when they provided directly relevant evidence on ceria facets, oxygen vacancies, Ce^3+^/Ce^4+^ redox behaviour, hydroxyl and water interactions, metal–ceria interfaces, or reaction-induced structural evolution. For quantitative comparison, studies were required to report the reaction conditions and at least one catalytic-performance metric. Studies focused exclusively on unrelated reactions, CO_2_ capture without hydrogenation, catalyst preparation without relevant catalytic or mechanistic information, inaccessible full texts, and duplicate reports were excluded from the quantitative comparison. Non-hydrogenation studies retained in the main text were used only as supporting evidence for ceria defect chemistry, interfacial structure, or characterization methodology and were not treated as direct CO/CO_2_ hydrogenation performance studies. Existing reviews have generally addressed individual products, metals, or catalyst architectures, including Cu/ZnO-based methanol synthesis, tandem catalysis, and Ni/CeO_2_ methanation [8,9,10,11,12,27]. To clarify the position of the present review relative to representative previous studies, Table 1 compares five influential reviews in terms of their scope, principal emphasis, and key limitations. These studies cover ceria defect chemistry, morphology and facet effects, noble-metal/ceria interfaces, bifunctional CO and CO_2_ hydrogenation, and Ni/ceria methanation.

Taken together, these studies have established important foundations in ceria defect chemistry, morphology control, metal–ceria interactions, bifunctional catalyst design, and Ni-centred methanation. However, the comparison indicates that previous reviews remain largely product-, metal-, or architecture-specific. An integrated framework linking defect location and characterization evidence with reaction pathways, product selectivity, and stability across pristine CeO_2_, single-metal, bimetallic, and mixed-oxide/CeO_2_ systems has not yet been systematically consolidated. The present review addresses this gap through a function-oriented structure–mechanism–performance framework. We therefore begin with pristine CeO_2_ as a dynamic redox, acid–base, and defect platform before discussing metal-modified and composite CeO_2_ catalysts.

## 2. Pristine Ceria

Starting from pristine CeO_2_ provides a basis for understanding the behaviour of composite catalysts. Although nanorods, nanocubes and polyhedra are commonly associated with different exposed facets, the actual reaction surface may reconstruct under reducing atmospheres, CO_2_/H_2_O adsorption and metal contact [22,23,31,32,33,34,35,36,37]. Therefore, morphology and the measured Ce^3+^ fraction should not be directly equated with the number of reactive oxygen vacancies.

### 2.1. Fundamental Role of Pristine CeO_2_: Not an Inert Support but a Reconfigurable Redox Platform

The primary role of pristine CeO_2_ is to provide a tunable redox, acid–base and defect environment for CO_2_ adsorption and intermediate stabilization, rather than to perform deep hydrogenation independently [20,28,31,37,38].

**Figure 1:**
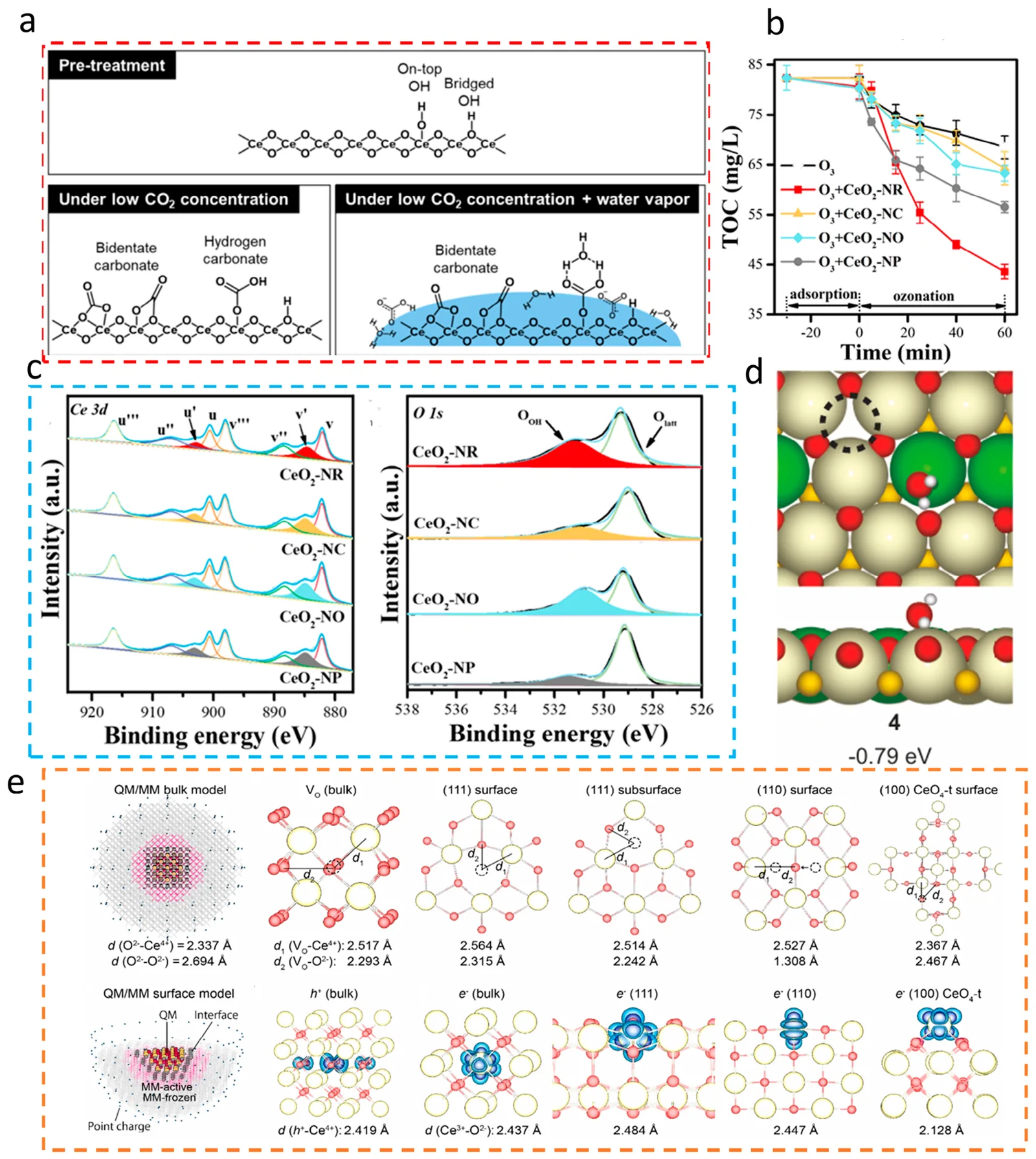
(**a**) Image of CO_2_ adsorption on CeO_2_ under low-concentration conditions. Reprinted with permission from Ref. [31]. Copyright 2024, with permission from Elsevier. (**b**) The time-dependent phenol degradation curves the kinetics of O_3_ decomposition. (**c**) Ce 3d and O 1s XPS spectra of different CeO_2_ samples. (**b**,**c**) Reprinted from Ref. [33], Copyright (2024), with permission from Elsevier. (**d**) Water adsorption at the CeO_2_(111) surface in the presence of a subsurface oxygen vacancy, together with the corresponding Ce^3+^ distribution on the reduced surface. Reproduced from Ref. [35], under CC BY 4.0 license. (**e**) Preferential localization of electrons on surfaces of CeO_2_ calculated with hybrid QM/MM embedded-cluster models. Reproduced from Ref. [37], under CC BY 4.0 license.

Low-concentration CO_2_ adsorption studies show that CeO_2_ forms bidentate carbonate, bicarbonate, monodentate carbonate and polydentate carbonate species even at 0.04 vol% CO_2_. Water vapour increases CO_2_ uptake by approximately 20% through hydrogen-bond-assisted adsorption, as shown in Figure 1a [31]. These observations indicate that surface hydroxyl groups and adsorbed water modulate the initial CO_2_ adsorption configuration via hydrogen-bonding interactions.

### 2.2. Facets and Morphology: (111)/(100)/(110) Determine Oxygen Vacancies, OSC, and Surface Reconstruction

Morphology control of CeO_2_ is the first structural variable for understanding its synergistic catalysis. Classical morphology-selective synthesis studies have shown that CeO_2_ nanopolyhedra, nanorods, and nanocubes expose different facet combinations: polyhedra mainly expose (111)/(100), nanorods mainly expose (110)/(100), and nanocubes mainly expose (100). These facet differences directly affect oxygen storage/release behavior; at 400 °C, nanorods and nanocubes involve both surface and bulk oxygen storage/release, whereas nanopolyhedra are more biased toward surface oxygen storage/release [20]. Thus, the “oxygen vacancy concentration” of CeO_2_ is not a single value independent of facet and spatial location, but is coupled with exposed facets, particle size, and reducible depth. However, the nominal morphology of CeO_2_ does not equal the real reaction surface. Using CO chemisorption probes combined with single-crystal calibration, Yang et al. directly observed surface reconstruction of rod-shaped CeO_2_ nanoparticles, which preferentially expose (111) nanofacets under reducing conditions [22]. In situ aberration-corrected STEM imaging further visualizes the formation of jagged reconstructed structures on CeO_2_(100) surfaces at 600 °C under low oxygen partial pressure. Particle swarm optimization combined with density functional theory (DFT) calculations rationalize these observations by identifying the thermodynamically stable surface structures across different Ce/O stoichiometries [36].

Facet effects are reflected not only in oxygen storage/release but also in the directionality of catalytic reactions and metal-support interactions. For example, in CuO_x_/CeO_2_ CO oxidation, CeO_2_ nanospheres exposing (111)/(100) and nanorods exposing (110)/(100) exhibit markedly different interaction strengths and interfacial oxygen migration abilities with CuO_x_ clusters, indicating that CeO_2_ facets further determine the dispersion, valence, and interfacial activity of supported metal oxides [39]. Vilé et al. proposed that CeO_2_ exhibits “opposite facet sensitivity” in hydrogenation and oxidation: (100)-terminated nanocubes favor CO oxidation, whereas (111)-dominated polyhedra are superior in acetylene hydrogenation. The key explanation is that different reactions have different requirements for oxygen vacancies and lattice oxygen; oxidation reactions often rely on surfaces that more readily form/replenish oxygen vacancies, whereas certain hydrogenation reactions may require low-defect, fully oxygen-coordinated surfaces to stabilize key intermediates [23]. This directly refutes the linear narrative of “more oxygen vacancies are better.”

This non-linear relationship is also corroborated in other environmental reactions. As shown in Figure 1b, studies on CeO_2_ with different morphologies for catalytic ozonation found that CeO_2_ nanorods exposing (110)/(100) facets exhibit the highest ozone decomposition and pollutant degradation activity, and the Frenkel-type oxygen vacancy density correlates linearly with the O_3_ decomposition rate [33]. Although this reaction is not CO_2_ hydrogenation, it demonstrates that CeO_2_ facets control Frenkel defects and surface reactive oxygen generation; these structural descriptors can be transferred to explain the formation of oxygen vacancies, hydroxyls, and CO_2_ adsorption species in CO_2_ hydrogenation.

Surface chemical analysis further shows that the Ce^3+^ fraction and hydroxyl-related O 1s component vary with CeO_2_ morphology. In Figure 1c, The surface Ce^3+^ fractions are approximately 16.7–24.4%, while the hydroxyl-related signal follows the order CeO_2_-NO > CeO_2_-NR > CeO_2_-NC > CeO_2_-NP [33]. These data indicate that morphology changes both the oxidation-state distribution and the hydroxyl environment of CeO_2_.

Therefore, the pristine CeO_2_ section should emphasize “facet-defect-reaction type matching” rather than simply ranking (100), (110), or (111). For CO_2_ hydrogenation, (110)/(100) often favor oxygen vacancy formation and CO_2_ activation, while (111) may provide a more stable and reversible adsorption environment; however, real catalysts reconstruct under reaction conditions, and the final active sites should be confirmed by in situ/operando characterization [20,22,23,33,36].

### 2.3. CO_2_ Adsorption/Desorption: Reversible Adsorption Matters More than Maximum Uptake

The CO_2_ adsorption capacity of CeO_2_ is often used as a prerequisite to explain CO_2_ hydrogenation performance, but one must distinguish between “being able to adsorb CO_2_” and “being able to convert adsorbed CO_2_ to the target product.” These adsorption results further show that water and hydroxyls regulate the identity and reversibility of carbonate species rather than simply competing with CO_2_. On-top hydroxyls and adsorbed water promote bicarbonate formation, whereas bridging hydroxyls are less reactive [31]. This coupling is relevant to subsequent formate formation in CO_2_ hydrogenation.

However, higher CO_2_ uptake does not necessarily correspond to better catalytic performance. Studies on the effect of exposed facets and oxidation state on CO_2_ adsorption/desorption show that cubic CeO_2_, mainly exposing (001)/(100)-like surfaces, has higher oxygen vacancy concentration and stronger CO_2_ adsorption; reduction treatment increases CO_2_ uptake, but part of the adsorbed CO_2_ is difficult to desorb, leading to irreversible adsorption. In contrast, polyhedral CeO_2_, despite lower uptake, can achieve reversible CO_2_ adsorption/desorption cycling under both oxidizing and reducing conditions. DFT results show that the monodentate/bidentate adsorption energy of CO_2_ on CeO_2_(111) is about −0.56 eV, whereas on CeO_2_(100) the bidentate adsorption energy is about −1.10 eV; after oxygen vacancy formation on CeO_2_(100), the adsorption energy can be as strong as −3.40 eV, easily causing strong adsorption and difficult desorption [34]. This is particularly critical for CO/CO_2_ hydrogenation catalyst design. For RWGS or methanol synthesis, overly strong carbonate may act as an inert overlayer, reducing the availability of H_2_ activation sites or metal–oxide interfaces; for methanation, overly strong CO_2_ adsorption may also cover the surface with carbonates/bicarbonates, limiting subsequent hydrogenation. Conversely, too weak adsorption is insufficient to polarize CO_2_. Therefore, the goal of CeO_2_ surface design is not to maximize CO_2_ uptake, but to obtain “convertible, desorbable, and regenerable” CO_2_ adsorption species [31,34].

### 2.4. Hydroxyls, Water, and Defects: Ce^3+^-Neighboring Sites Determine Initial Adsorption Events

CO_2_ hydrogenation inevitably produces H_2_O, and the feed gas, low-concentration CO_2_ capture, or real flue gas often contains water. Thus, the interaction of pristine CeO_2_ with water/hydroxyls should be considered an important part of its synergistic function. AFM/DFT studies on water adsorption symmetry breaking at near-surface defects on CeO_22−x_(111) show that water molecules exhibit an asymmetric “boomerang-like” feature near defective regions, rather than the previously assumed symmetric triangular feature; as shown in Figure 1d, DFT further indicates that Ce^3+^ sites adjacent to subsurface oxygen vacancies are thermodynamically more favorable for water adsorption, with molecular adsorption energy on Ce^3+^ of about −0.79 eV, stronger than on some Ce^4+^-neighboring sites [35]. This suggests that subsurface defects in CeO_2_, although not necessarily directly exposed, influence water adsorption and hydroxyl generation by modifying the Ce^3+^ distribution and local symmetry.

This understanding connects directly with low-concentration CO_2_ adsorption studies. Water vapor does not simply compete for CO_2_ sites; it can enhance CO_2_ adsorption via hydrogen bonding and promote bicarbonate formation; however, in some CO_2_ hydrogenation catalysts, water may also promote OH accumulation, support sintering, or block active interfaces. Therefore, water should not be universally described as a poison or a promoter; rather, its different roles in initial CO_2_ adsorption, hydroxyl formation, oxygen vacancy repair, product desorption, and long-term stability should be distinguished [31,34,35,37].

At metal–CeO_2_ interfaces, an appropriate hydroxyl coverage can provide Lewis basicity and assist proton transfer, whereas excessive hydrophilicity or strongly binding vacancies may cause OH accumulation, irreversible carbonate formation and interface blocking [31,34,35].

### 2.5. Defect Electronic Structure: From “One Oxygen Vacancy Corresponds to Two Ce^3+^” to Local Heterogeneity

The traditional description of CeO_2_ defect chemistry often states that “formation of one oxygen vacancy is accompanied by reduction of two Ce^4+^ to Ce^3+^.” This global charge compensation relationship holds stoichiometrically, but a combined theoretical-experimental study in *JACS* 2025 showed that it does not always hold at the nanoscale local level. As shown in Figure 1e, QM/MM defect calculations, synchrotron XPS, and Monte Carlo simulations reveal that electrons tend to localize/segregate on CeO_2_ surfaces and can even overcome the trapping of electrons by bulk oxygen vacancies; surface oxygen vacancies trap electrons more readily than bulk vacancies, so the surface Ce^3+^/oxygen vacancy ratio can be higher than in the bulk. This electron surface segregation is more pronounced at lower reduction degrees and for smaller nanoparticles [37].

This understanding is very important for interpreting CO/CO_2_ hydrogenation catalysts. Many reports use XPS Ce^3+^ fraction, Raman D-band, or H_2_-TPR peak area to represent “oxygen vacancy concentration,” but these indicators are often mixed responses from surface, near-surface, and bulk defects; the defects that actually participate in CO_2_ adsorption, H_2_O dissociation, metal anchoring, or H spillover are typically local surface defects and electron-rich sites [35,37]. Therefore, when comparing the activities of Cu/CeO_2_, Ni/CeO_2_, Pd/CeO_2_, or Ru/CeO_2_, one should not merely compare total Ce^3+^ fractions, but should focus on defect location, electron localization degree, facet environment, and metal–oxide contact geometry.

Importantly, these characterization techniques should be regarded as complementary rather than interchangeable probes of oxygen vacancies. Ce 3d XPS primarily reflects the near-surface Ce^4+^/Ce^3+^ redox-state distribution, whereas O 1s XPS provides semi-quantitative information on lattice oxygen, defect-associated oxygen and hydroxyl or adsorbed oxygen species; neither measurement alone can determine the absolute vacancy concentration or unambiguously distinguish surface from subsurface vacancies [33]. Raman spectroscopy probes lattice disorder through perturbation of the CeO_2_ F_2_g mode and defect-induced vibrational bands, providing an averaged structural signature of vacancy-related distortion with limited spatial resolution [17]. EPR is sensitive to paramagnetic vacancy-related centers, such as electron-trapped F^+^ centers and adsorbed superoxide species, but is unable to detect diamagnetic or electronically silent vacancies [17]. H_2_-TPR mainly measures the reducibility and oxygen mobility of surface and bulk domains through reduction temperatures and hydrogen consumption, whereas CO_2_-TPD and H_2_-TPD primarily probe the strength and distribution of basic CO_2_-adsorption sites and hydrogen adsorption or spillover, respectively; these techniques therefore provide indirect descriptors rather than direct measurements of vacancy concentration [40]. In situ or operando XPS, XAS, Raman, EPR and DRIFTS can further monitor the reaction-induced evolution of Ce valence, local coordination, vacancy-associated species and adsorbed intermediates under realistic reaction conditions [41,42]. Accordingly, oxygen vacancies should be reported as method-specific observables—including surface Ce^3+^ enrichment, lattice-disorder signatures, paramagnetic centers, reducibility or oxygen mobility, and vacancy-associated adsorption sites—rather than as a single, uniform vacancy population [16,17].

Overall, pristine CeO_2_ should be viewed as a dynamic platform in which facet structure, defect location, Ce^3+^ distribution, hydroxyl chemistry and surface reconstruction jointly determine CO_2_ adsorption and intermediate stabilization [20,22,31,34,35,37]. These descriptors should therefore be evaluated under reaction conditions rather than inferred from a single ex situ measurement.

Mechanistic assignments throughout this review are made according to the proximity of each observation to catalytic turnover. Species detected under reaction conditions by operando spectroscopy, isotopic tracing, or transient kinetic measurements provide the strongest basis for identifying reaction intermediates and participating sites. Changes in activity or selectivity that accompany variations in defect concentration, exposed facets, or metal–ceria contact are interpreted as structure–performance relationships, but they are not considered sufficient on their own to establish causality. DFT calculations and microkinetic models are used to evaluate the plausibility of proposed pathways and to identify kinetically demanding steps, while recognizing their dependence on surface models, adsorbate coverage, and reaction conditions. When the available evidence is indirect or system-specific, the corresponding interpretation is stated as proposed, consistent with, or associated with, rather than universally established. This approach is adopted in the following sections to distinguish reaction intermediates observed during operation from structural descriptors and model-based mechanistic interpretations.

Vacancy concentration alone is not a transferable predictor of selectivity. Surface and bulk vacancies differ in their electron-localization behaviour, and a measured Ce^3+^ fraction may therefore contain contributions from electronically and chemically distinct defects [37]. Facet-controlled studies further show that reduced cubic ceria can bind CO_2_ strongly enough to form poorly reversible carbonate species, whereas predominantly (111)-terminated ceria can provide more reversible adsorption [34]. Even within Pd/CeO_2_ methanol catalysts, the support with the largest vacancy population did not exhibit the highest vacancy-normalized turnover frequency, demonstrating that vacancy reactivity depends on local coordination and formation energy rather than defect number alone [43].

The relevant descriptor is therefore a reactive site ensemble, comprising a vacancy or reducible Ce site, an adjacent metal site, and an interfacial geometry that permits intermediate turnover. Facets regulate this ensemble by controlling vacancy stability, metal anchoring, and local oxygen mobility. Metal nuclearity then determines whether CO* desorbs, undergoes sequential hydrogenation, or remains available for C–C coupling. This distinction is illustrated by Rh/CeO_2_, where interfacial vacancy density mainly governs activity, whereas Rh particle size governs the CO/CH_4_ product split [44]. Consequently, high vacancy density, high Ce^3+^ content, or strong metal-support interaction should not be treated as universal design targets.

## 3. Ce-Based Mixed Oxides

### 3.1. Single-Metal-Modified Ceria

The core differences among single-metal/CeO_2_ systems arise from the intrinsic affinities of the metals toward H_2_, CO*, and oxygenated intermediates. Single-metal/CeO_2_ systems are distinguished here according to the dominant function of the metal. Cu mainly regulates oxygenate formation and RWGS, Ni, Co and Ru promote deep hydrogenation, whereas Pd, Rh and Au broaden the selectivity range through changes in metal nuclearity and interfacial coordination [45,46,47,48,49,50,51,52]. This section also cautions against two common over-interpretations. First, a higher Ce^3+^ fraction is not directly equivalent to higher CO_2_ hydrogenation activity, because the metal sites may no longer effectively dissociate H_2_. Second, strong metal-support interaction is not always synonymous with high stability, as excessive encapsulation may sacrifice exposed metal sites. More reliable judgment should combine metal-normalized TOF, interface length or exposed sites, reaction orders, and in situ intermediate evolution.

Within the catalyst-specific discussion below, “observed under reaction conditions” is reserved for species or structural changes detected by operando or in situ spectroscopy, isotope tracing, or transient methods. Ex situ characterization is used to define pre- or post-reaction structures and to establish structure-performance correlations. DFT and microkinetic results are discussed as model-dependent tests of pathway feasibility. Accordingly, site assignments are termed experimentally supported only when multiple operation-relevant observations converge; otherwise, they are described as proposed models or interpretations consistent with available evidence.

#### 3.1.1. Cu-Modified Ceria

##### Cu/CeO_2_ Interface: From Cu Modification to Bifunctional Active Sites

The Cu–CeO_2_ contact has been proposed as a bifunctional reaction region rather than a conventional support boundary [53,54]. Experimental and theoretical studies suggest that Cu-containing sites can contribute to H_2_ dissociation and hydrogen transfer, whereas defective CeO_2_ can stabilize activated CO_2_ and oxygenated intermediates. The relative contribution of these functions is associated with the competition between methanol formation and RWGS, but its magnitude depends on Cu nuclearity, interfacial geometry, defect location and reaction atmosphere [41,46]. Related defect–interface concepts reported for other oxide systems provide a broader methodological framework, but the Cu–CeO_2_ interface remains the primary focus here [27,55,56].

**Figure 2:**
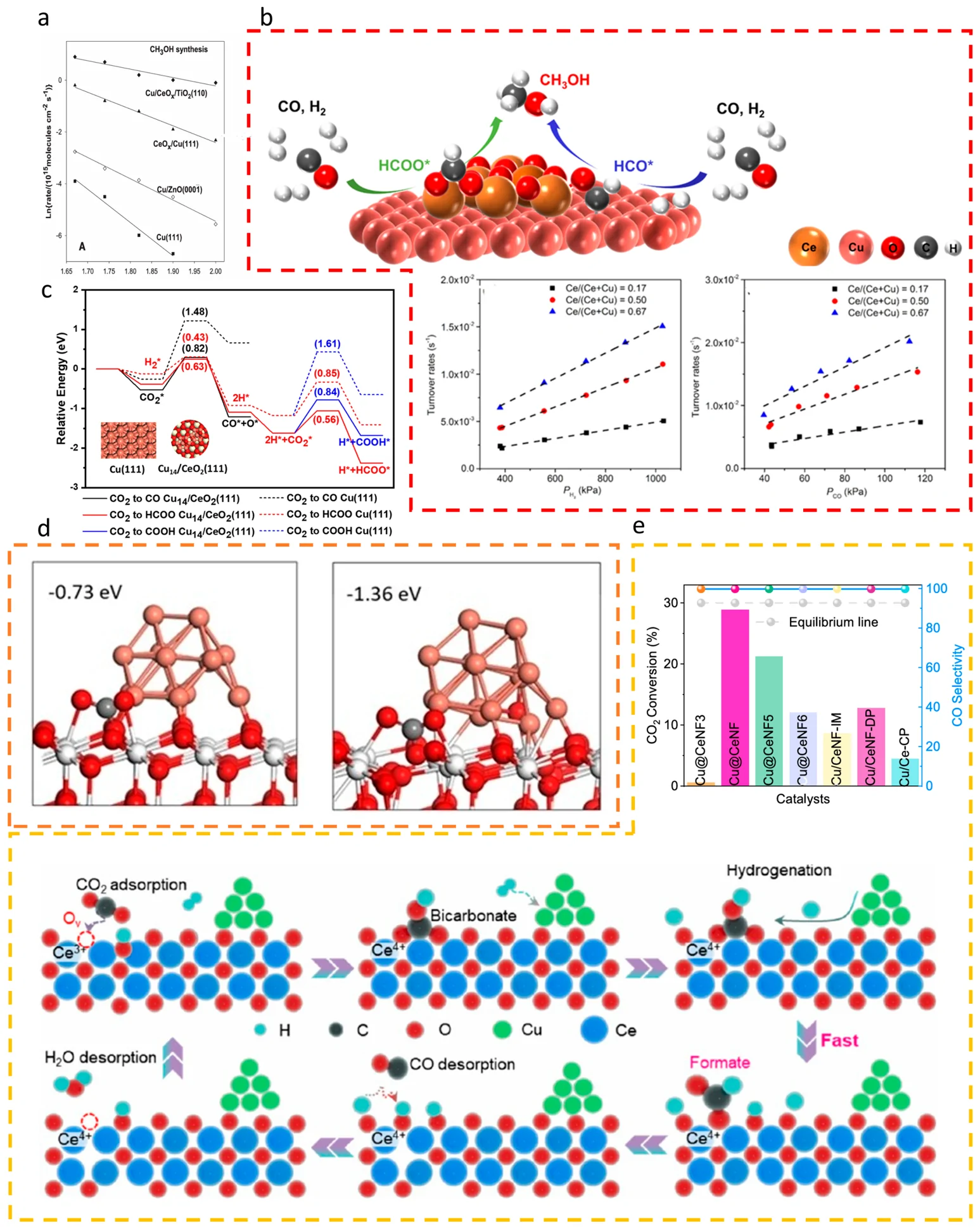
(**a**) Arrhenius plot for methanol synthesis on Cu (111), a 0.2 ML of Cu on ZnO (0001^−^), a Cu (111) surface covered 20% by ceria, and a 0.1 ML of Cu on a TiO_2_ (110) surface pre-covered 15% with ceria. In a batch reactor, the catalysts were exposed to 0.5 atm of CO_2_ and 4.5 atm of H_2_. The reported values are steady-state rates measured at 600, 575, 550, 525, and 500 K. Reproduced from Ref. [49], Copyright (2014), with permission from AAAS. (**b**) Proposed mechanisms of methanol synthesis over Cu/CeO_2_ catalysts and turnover rates for CH_3_OH formation as a function of H_2_ and CO partial pressures. Reprinted from Ref. [58], Copyright (2022), with permission from Elsevier. (**c**) Potential energy diagrams for the initial CO_2_ hydrogenation steps on Cu_14_/CeO_2_ (111) and Cu (111) based on DFT calculations. Reprinted from Ref. [41], under CC BY 4.0 license. (**d**) Optimized structure of CO_2_ adsorption on Cu–CeO_2_ and Cu–CeO_2_–V. CO_2_ adsorption energy of different structure. Reproduced from Ref. [40], Copyright (2025), with permission from Wiley-VCH. (**e**) Proposed RWGS reaction mechanism over Cu@CeNF and the corresponding CO_2_ conversion and CO selectivity for Cu@CeNF-based catalysts. Reproduced from Ref. [59], Copyright (2026), with permission from American Chemical Society.

The most representative evidence comes from the CeO_x_/Cu(111) model catalyst in Figure 2a: at 575 K, the methanol synthesis TOF on CeO_x_/Cu(111) is estimated to be at least 1.3 molecules site^−1^ s^−1^, about 200 times that of Cu(111) and 14 times that of Cu/ZnO(0001); the apparent activation energy also decreases from 25 kcal mol^−1^ on Cu(111) to 12 kcal mol^−1^ [49]. Under the investigated model-catalyst conditions, this comparison provides evidence that reducible oxide–metal interfaces can open CO_2_ activation routes distinct from those available on metallic Cu surfaces. AP-XPS and IRRAS further detected formate and carboxylate species, among which the unstable CO_2_ᵟ^−^/carboxylate is more likely to be a dynamic intermediate toward methanol, while stable formate is closer to a surface overlayer or a bypass intermediate. This conclusion is also summarized in reviews on metal–oxide/metal–carbide interfaces for CO_2_ activation [49,54,57].

##### Differentiation of CO and CO_2_ Hydrogenation Pathways: Same Interface, Different Carbon Sources

Cu/CeO_2_ exhibits significantly different active-site requirements in CO hydrogenation versus CO_2_ hydrogenation. Under CO/H_2_ conditions, the Cu-CeO_2_ interface tends to act as the active center for direct CO hydrogenation to methanol, whereas under CO_2_/H_2_ conditions, there is a more complex competition between the metallic Cu surface and the formate coverage induced by CeO_2_. Van de Water et al. found that during CO/H_2_ feed, transient CO_2_ formation precedes methanol production, and CO_2_ can then re-adsorb as carbonate and formate, causing gradual deactivation in different zones of the catalytic bed [45]. They accordingly proposed that the active site for steady-state CO hydrogenation to methanol is the “interface between defective CeO_2−x_ and highly dispersed Cu particles,” where Cu exists in both Cu^0^ and Cu^+^ states; CO_2_ acts as a poison for this interface, and a small amount of CO_2_ added to CO/H_2_ can reduce activity through carbonate/formate accumulation.

Ren et al. further clarified the paired-site feature of CO hydrogenation under real reaction conditions. Isolated Cu, CeO_2_, or physical mixtures of Cu + CeO_2_ produce almost no methanol at 493 K, whereas Cu/CeO_2_ catalysts can generate CH_3_OH with high selectivity. In Figure 2b, as the Ce content increases from 17% to 67%, the TOF of CH_3_OH formation based on surface Cu^0^ increases from 4.32 × 10^−3^ s^−1^ to 1.2 × 10^−2^ s^−1^, with methanol carbon selectivity above 97%; The monotonic relationship with the surface Ce^3+^/Cu^0^ ratio is consistent with a contribution from Cu^0^–Ce^3+^ paired sites, although the correlation alone does not establish that these pairs are the only active structures [58]. Reaction orders for CO and H_2_ in methanol formation are about 0.80 and 0.85, respectively, indicating that both participate in kinetically relevant steps. CO-DRIFTS shows a CO adsorption peak at about 2098 cm^−1^ assigned to interfacial metallic Cu sites, and H_2_-TPD reveals that H species can distribute between interfacial Cu and Ce^3+^ sites; operando DRIFTS captured HCO*, HCOO*, and H_3_CO*, with H_3_CO* formation considered closely related to the kinetic step of methanol generation.

In contrast to CO hydrogenation, the Hensen group’s comparison of CO_2_/CO hydrogenation over conventional Cu/CeO_2_ showed that strong CO_2_ adsorption can block the interfacial CO hydrogenation pathway, so that methanol mainly comes from CO_2_ hydrogenation rather than CO intermediates. They developed a CO_2_-N_2_O combined titration method: first using CO_2_ to block CeO_2_ oxygen vacancies, then using N_2_O to distinguish contributions from metallic Cu sites and CeO_2_ vacancies, thus avoiding the misestimation of oxygen vacancies as Cu surface area [41]. The results indicate that Cu-CeO_2_ interaction significantly improves Cu dispersion and gives Cu/CeO_2_ higher methanol selectivity than Cu/SiO_2_, but this improvement is not due to a significant increase in methanol formation rate, but rather to the suppression of RWGS. In Figure 2c, DFT and in situ IR show that the formate formation barrier on Cu_14_/CeO_2_ is 0.56 eV, lower than the direct CO_2_ dissociation barrier of 0.82 eV and the carboxyl formation barrier of 0.84 eV. Together, the calculated barriers and in situ infrared observations support a formate-favoured surface chemistry under the investigated conditions, which may suppress the CO-mediated RWGS route when formate coverage becomes sufficiently high. Oxygen Vacancies, Ce^3+^, and Cu Valence: Three Handles for Selectivity Control.

The key to Cu-CeO_2_ synergy is not simply increasing Cu loading, but simultaneously regulating oxygen vacancy concentration, Ce^3+^ stability and the Cu^0^/Cu^+^ ratio. Oxygen vacancies serve as CO_2_ adsorption and activation sites; Ce^3+^ reflects the reduction degree and electron buffering capacity of CeO_2_; Cu^0^ mainly undertakes H_2_ dissociation and CO adsorption; and Cu^+^ is more favorable for stabilizing oxygenated intermediates such as formate and methoxy [46,60,61,62,63]. Different target products require different optimal interfacial electronic structures: for methanol, CO_2_ adsorption and oxygenate hydrogenation ability should be enhanced while avoiding excessive CO desorption; for CO, formate decomposition or CO desorption should be accelerated to avoid deep hydrogenation. The facet-dependent Cu-Ce interface and Cu^0^-Oᵥ synergy observed in liquid-phase hydrogenation of unsaturated aldehydes over Cu/CeO_2_ also serves as a side proof of “oxygen vacancy-metal site bifunctional division” [64,65].

The valence and dispersion state of Cu species themselves remain critical for the selectivity of single-metal Cu/CeO_2_. Studies on Cu loaded on CeO_2_ with different morphologies show that exposed facets alter the Cu^+^/Cu^0^ ratio, oxygen vacancy density, and CO_2_ adsorption strength, thereby modulating the competition between methoxy, formate, and CO* [60,61]. Recent studies on dissociated hydrogen further indicate that hydrogen migration between Cu sites and CeO_2_ defects contributes to methoxy formation and methanol desorption, supporting the view that Cu/CeO_2_ is a dynamic Cu–Ce–Oᵥ interface [63].

The synergistic introduction of Silicalite-1 zeolite with CeO_2_ exemplifies the stabilization of Cu species by “interfacial microenvironment.” Xu et al. co-introduced pure-silica S-1 zeolite and CeO_2_ into Cu catalysts, stabilizing small-sized CeO_2_ around Cu to construct a Cu-CeO_2_ interface rich in Cu^+^ and oxygen vacancies. CuCe/S-1 achieved a methanol STY of 87.23 g kg_Cu^−1^ h^−1^ in CO_2_-to-methanol, about 5 times that of Cu/CeO_2_; TOF was 4.16 h^−1^, about 3 times that of Cu/CeO_2_; apparent activation energy decreased from 37.08 kJ mol^−1^ for Cu/CeO_2_ to 24.69 kJ mol^−1^ for CuCe/S-1 [40]. The Ce^3+^ fraction of CuCe/S-1 was 56%, higher than 44% for Cu/CeO_2_, and the CO_2_-TPD desorption peak shifted from 251 °C for Cu/CeO_2_ to 315 °C. In Figure 2d, DFT shows that the adsorption energy of CO_2_ on a normal Cu-Ce interface is −0.73 eV, while the introduction of an oxygen vacancy reduces it to −1.36 eV, indicating that the oxygen vacancy-rich interface decisively enhances CO_2_ adsorption.

##### Cu-Oᵥ Interface in RWGS: Steering Synergy Toward CO Selectivity

When the reaction target shifts from methanol to CO, the design logic of Cu/CeO_2_ must switch from “stabilizing and hydrogenating oxygenated intermediates” to “rapidly generating and desorbing CO after CO_2_ activation.” Studies on spatially confined Cu/CeO_x_ show that, under similar Cu loading, particle size, and dispersion, CeO_2_ promotes CO_2_-to-CO conversion much more effectively than SiO_2_. Cu nanoparticles lower the low-temperature reduction temperature of CeO_2_, allowing oxygen vacancies to form below 300 °C; Cu/CeO_x_ maintains 100% CO selectivity near equilibrium conversion at 300 °C and atmospheric pressure, and its activity is about 4 times that of structurally similar Cu/SiO_2_ [66]. In situ XRD/XAS/FTIR observed Cu^+^-CO formation together with oxygen vacancies, indicating that RWGS is not a single-metal Cu reaction, but is accomplished by Cu-promoted H_2_ activation and CeO_2−x_-provided CO_2_ activation sites.

Combustion-synthesized Cu-CeO_2_ solid solutions also show synergistic RWGS capability between CeO_2_ surface oxygen and Cu species. 1 wt% Cu/CeO_2_ produced CO as the sole carbon product in the 150–600 °C range, with about 70% CO_2_ conversion at 600 °C, and remained stable over 1200 min with carbon deposition below 0.05% [67]. CeO_2_@Cu self-combustion systems based on micron-sized Cu powder and thermally reconstructed Cu/CeO_2_ interface oxygen vacancy systems also demonstrate that Cu-O-Ce interfacial oxygen defects significantly enhance CO formation in RWGS [47,48]. Furthermore, comparative studies on transition-metal-doped CeO_2_ show that Cu-doped CeO_2_ can maintain 100% CO selectivity, but CO_2_ conversion is relatively limited, indicating that Cu doping alone is insufficient to replace a high-density, regenerable interfacial oxygen vacancy design [68].

Going a step further, the Cu@CeNF hollow-fiber structure in ACS Catalysis 2026 explicitly targets “high-density Cu-Oᵥ interfaces” for RWGS. Electrospun Cu nanoparticles anchored on oxygen vacancy-rich hollow CeO_2_ fibers create numerous C_n_-Oᵥ synergistic sites. In Figure 2e, Cu@CeNF achieved 28.9% CO_2_ conversion and 99.9% CO selectivity at 350 °C, compared with only 4.5% for coprecipitated Cu/Ce-CP; at 300 °C, Cu@CeNF gave 12.9% CO_2_ conversion, about 8.6 times that of Cu/Ce-CP, and remained stable for 228 h at 300 °C and GHSV = 60,000 mL g^−1^ h^−1^ [59]. XPS showed that the Ce^3+^ fraction of Cu@CeNF was 15.6%, higher than 9.2% for Cu/Ce-CP, and increased to 22.3% after reduction; DRIFTS indicated that CO_2_ first forms bicarbonate near oxygen vacancies/hydroxyls, then reacts with H dissociated from Cu sites to generate formate, and finally produces CO via *HCOO decomposition.

A recent study on Hollow-sphere CeO_2_-supported Cu provides a complementary example in which structural confinement improves Cu dispersion, mass transfer and the accessibility of Cu–CeO_2_ interfacial sites. Zhang et al. highly dispersed Cu on hollow-sphere CeO_2_ to construct a Cu-S-Ce catalyst; this structure enhances mass transfer, reactant enrichment, and active-site exposure via cavities and high surface area, while also shortening electron migration distances and stabilizing oxygen vacancies through strong Cu-CeO_2_ interaction. At 500 °C, the TOF_Cu of Cu-S-Ce reached 4.56 s^−1^, with CO selectivity near 100%, and good stability was maintained after 48 h of continuous operation at 600 and 700 °C [69].

In summary, the outstanding value of the Cu/CeO_2_ system lies in its ability to steer the same CO_x_ hydrogenation network toward different products through interfacial structure. For CO hydrogenation, Cu^0^-Ce^3+^ paired sites can activate CO and H_2_, and generate methanol via HCO*, HCOO*, H_3_CO*, and other intermediates. For CO_2_-to-methanol, the Cu-CeO_2_ interface enhances CO_2_ adsorption and formate/methoxy stabilization, but overly strong carbonate/formate coverage can also poison the CO hydrogenation interface or inhibit RWGS. For RWGS, C_n_-Oᵥ sites composed of oxygen vacancy-rich CeO_2_ and Cu nanoparticles promote CO_2_ adsorption, H_2_ activation, formate decomposition, and CO desorption, thus achieving high CO selectivity [41,45,46,58,59,66,67]. Therefore, the rational design of Cu/CeO_2_ catalysts should not rely solely on Cu loading, Cu particle size, or CeO_2_ surface area, but should focus on three structural descriptors: (1) the Cu-CeO_2_ interface length or interfacial site density; (2) the steady-state concentration of regenerable oxygen vacancies and Ce^3+^ under reaction conditions; and (3) the Cu^0^/Cu^+^ ratio and its balance between H_2_ dissociation, CO adsorption, and oxygenate intermediate stabilization [41,46,58,60,61,62,63,70]. To further improve CO_2_-to-methanol performance, two extremes must be avoided: on one hand, too weak a Cu-CeO_2_ interaction fails to activate CO_2_; on the other, too strong or too stable formate/carbonate coverage sacrifices turnover rates. Strategies such as W doping, zeolite confinement, small-sized CeO_2_ stabilization, and hollow-fiber/nanorod morphology construction that precisely tune the electronic structure and spatial proximity of the Cu-CeO_2_-Oᵥ interface represent key directions for moving this system from empirical preparation to designable catalysis [40,46,59,60]. The key to the Cu/CeO_2_ system does not lie in any single preparation method, but in the fact that the same Cu-Ce-Oᵥ interface can switch between methanol and CO through subtle adjustments in electronic structure and spatial proximity. Therefore, the Cu^0^/Cu^+^ ratio, regenerability of interfacial oxygen vacancies, formate coverage strength, and CO desorption capability should be understood within the same reaction coordinate, rather than compared in isolation for conversion or selectivity. From an industrial perspective, these structural descriptors directly map to core performance metrics for large-scale operation. Interfacial site density determines the intrinsic methanol productivity per reactor volume, a critical parameter for high space-velocity operation in commercial methanol plants [71]. Regenerable oxygen vacancies dictate catalyst service life and tolerance to water vapor and CO co-feeds, addressing the dominant deactivation pathways in long-term runs. The Cu^0^/Cu^+^ ratio governs product selectivity, which in turn reduces downstream separation costs associated with product purification. Current laboratory demonstrations typically report stability over 100–200 h under idealized feed conditions; bridging this gap to the industrial benchmark of 8000+ hours requires targeted engineering of interfacial stability and sintering resistance [71].

#### 3.1.2. Ni-Modified Ceria

##### Ni/CeO_2_ Interface: From CO_2_ Activation to Deep Hydrogenation—A Methanation Platform

Unlike Cu/CeO_2_, which is more commonly used for CO_2_-to-methanol or RWGS, the core reaction characteristics of Ni/CeO_2_ are strong H_2_ dissociation and deep hydrogenation capability, making it more prone to push the CO_2_ hydrogenation network toward CH_4_. A lateral comparison of first-row 3d transition metals on CeO_2_ nanorods showed that Ni/CeO_2_ exhibits the highest methanation activity and selectivity in CO_2_ hydrogenation, with a CH_4_ yield of 90.8% at 350 °C, significantly higher than 45.2% for Co/CeO_2_; this trend correlates with oxygen vacancies, Ce^3+^ concentration, and the intrinsic electronic structure of the 3d metals [51]. Structural studies on transition-metal-substituted CeO_2_ nanoparticles also indicate that transition metal incorporation into the CeO_2_ lattice alters local coordination, valence, and oxygen non-stoichiometry, providing a more fundamental materials chemistry basis for understanding Ni-Ce-O interface defects [72]. This shows that the synergy between Ni and CeO_2_ is not a simple superposition: Ni provides efficient H_2_ dissociation and CO/carbon intermediate hydrogenation sites, while CeO_2_ promotes CO_2_ adsorption/activation through Ce^4+^/Ce^3+^ cycling, oxygen vacancies, and moderate basic sites, thus constituting the bifunctional interface required for CO_2_ methanation [42,73,74].

**Figure 3:**
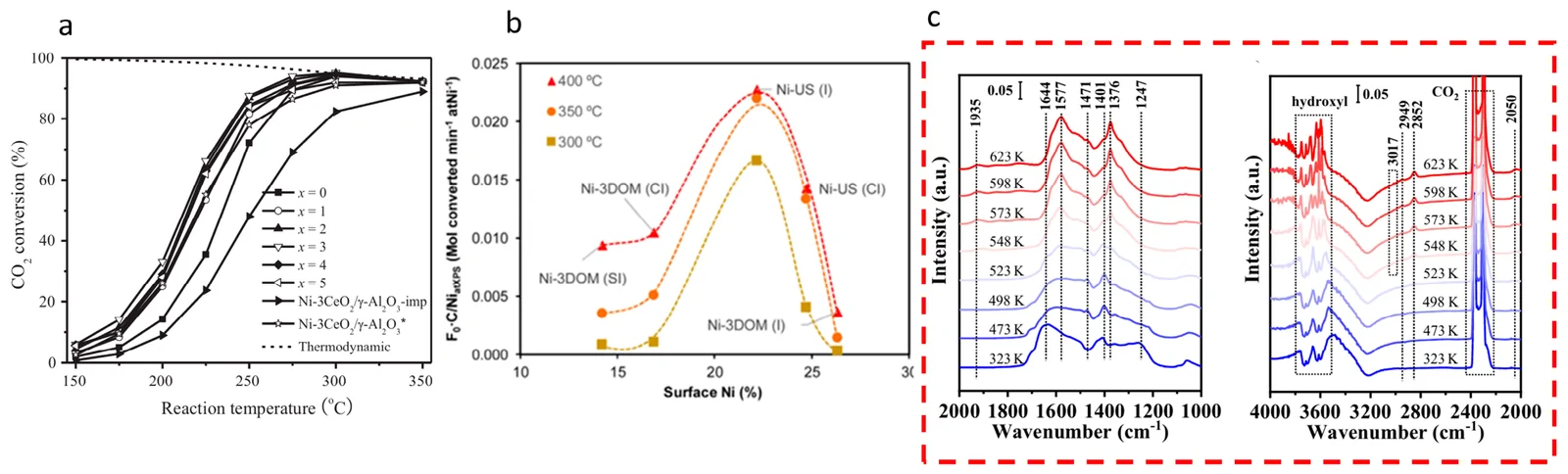
(**a**) The activity of the prepared Ni-x CeO_2_/γ-Al_2_O_3_, Ni-3CeO_2_/γ-Al_2_O_3_-imp and Ni-3CeO_2_/γ-Al_2_O_3_ samples for CO_2_ methanation. Reaction conditions: GHSV, 3000 mL·g_cat^−1^·h^−1^; H_2_/CO_2_, 4. Reprinted from Ref. [75], Copyright (2017), with permission from Elsevier. (**b**) Relationship between the activity per surface active site at different temperatures and the proportion of surface Ni^0^ determined by XPS for reduced catalysts. Reprinted from Ref. [74], Copyright (2020), with permission from Elsevier. (**c**) In situ FT-IR spectra of CO_2_ methanation reactions over Ni/CeO_2_-6M with different reaction temperatures. Reprinted from Ref. [76], Copyright (2023), with permission from Elsevier.

In early Ni-Ce-Al_2_O_3_ systems, the addition of CeO_2_ was shown to reduce Ni particle size, induce oxygen vacancies, and enhance low-temperature methanation. In Figure 3a, Nie et al. prepared NiO-xCeO_2_/γ-Al_2_O_3_ that exhibited excellent CO_2_ methanation performance in the 150–350 °C range; the optimal 3 wt% CeO_2_ sample reached near-equilibrium CO_2_ conversion at 300 °C with 100% CH_4_ selectivity [75].

Support differences further emphasize that CeO_2_ is not the only viable support, but its interfacial redox function is distinctive. Italiano et al. compared Ni/CeO_2_, Ni/Al_2_O_3_, and Ni/Y_2_O_3_ for CO/CO_2_ methanation and found that Ni/Y_2_O_3_ outperformed in stability and CO_2_ methanation, while Ni/CeO_2_ was prone to rapid deactivation due to carbon deposition; this suggests that Ni/CeO_2_ needs to overcome stability shortcomings through interfacial structure, oxygen vacancies, and Ni dispersion engineering [77].

##### Dual-Active-Site Model: Ni-CeO_2_ Interface Activates CO_2_, Ni^0^ Sites Activate H_2_

One of the most general structure-function relationships in Ni/CeO_2_ comes from Cardenas-Arenas et al. on the design of Ni-CeO_2_ contact modes. They clearly proposed two types of active sites: one is the CO_2_ chemisorption/dissociation site at the Ni-CeO_2_ interface, and the other is the H_2_ dissociation site on Ni^0^ particles. In Figure 3b, XPS results showed that when the surface Ni^0^ to NiO-CeO_2_ ratio is about 25%:75%, CO_2_ conversion is highest; too much Ni^0^ weakens CO_2_ activation sites, while too much Ni-CeO_2_ leads to accumulation of bicarbonate and other intermediates [74]. ^13^C^18^O_2_ isotope pulsing and in situ DRIFTS indicated that the optimal catalyst maintains a relatively “clean” surface during reaction, whereas excessive CO_2_ dissociation sites accumulate bicarbonate species.

Isotopic and DRIFTS studies further support this division of labor: the Ni–CeO_2_ interface activates CO_2_, Ni^0^ dissociates H_2_, and mobile lattice oxygen facilitates water removal [42]. Thus, the key limitation is not CO_2_ activation alone, but the coupled conversion of CO_2_-derived intermediates and removal of water from interfacial sites.

##### Pathway Divergence: Formate Pathway and RWGS + CO Hydrogenation Pathway Coexist

The mechanistic picture for Ni/CeO_2_ is best understood as a set of competing routes rather than a single universal sequence. In situ FTIR and DRIFTS studies detect carbonate, bicarbonate, formate, methoxy, and carbonyl species under methanation conditions, supporting the progressive hydrogenation of CO_2_-derived intermediates [73,76,78,79,80]. Isotopic experiments further indicate that CO-containing intermediates can participate in the reaction network, whereas the relative contributions of the formate and RWGS–CO hydrogenation routes vary with Ni particle size, defect environment, and operating conditions [42,76]. These observations establish which surface species are present during turnover, but they do not by themselves identify the rate-controlling step or demonstrate that one pathway dominates across all Ni/CeO_2_ catalysts.

DFT calculations provide a complementary interpretation of these observations. For the Ni/CeO_2_ model examined by Zhang et al., DFT calculations predict that Ni nanoclusters preferentially adsorb and activate CO_2_, whereas the modelled Ni–CeO_2_ interface can facilitate electron redistribution and hydrogenation of carbon-containing intermediates [78]. On the selected model surface, the calculated energy landscape favours the RWGS–CO hydrogenation sequence, CO_2_* → HOCO* → CO* → HCO* → H_2_CO* → CH_2_* → CH_3_* → CH_4_*, wand identifies H-assisted C–O bond cleavage in H_2_CO* as the most demanding step within that model. This result offers a mechanistic explanation for the coexistence of CO-, formate-, and methoxy-related species, but its quantitative applicability depends on the selected surface model and reaction conditions.

Experimental observations are also compatible with a formate-mediated route. By varying the CeO_2_ particle size and thereby modifying the Ni–CeO_2_ contact environment, Lin et al. found that Ni/CeO_2_-6M exhibited a CO_2_ conversion rate approximately three times higher than that of Ni/CeO_2_-12M at 548 K, and In situ FTIR in Figure 3c detected formate-, methoxy-, and carbonyl-related species during CO_2_ methanation, which is consistent with a formate-mediated route involving methoxy and carbonyl intermediates_4_ [76]. Ye et al. similarly observed formate, methoxy, and weak *CHO-related signals over Ni/CeO_2_-SGM, suggesting that highly dispersed Ni species and Ni–Ce–O structures facilitate the subsequent conversion of interfacial intermediates [73]. These results support the participation of formate-related species, although they do not exclude parallel CO-mediated pathways.

**Figure 4:**
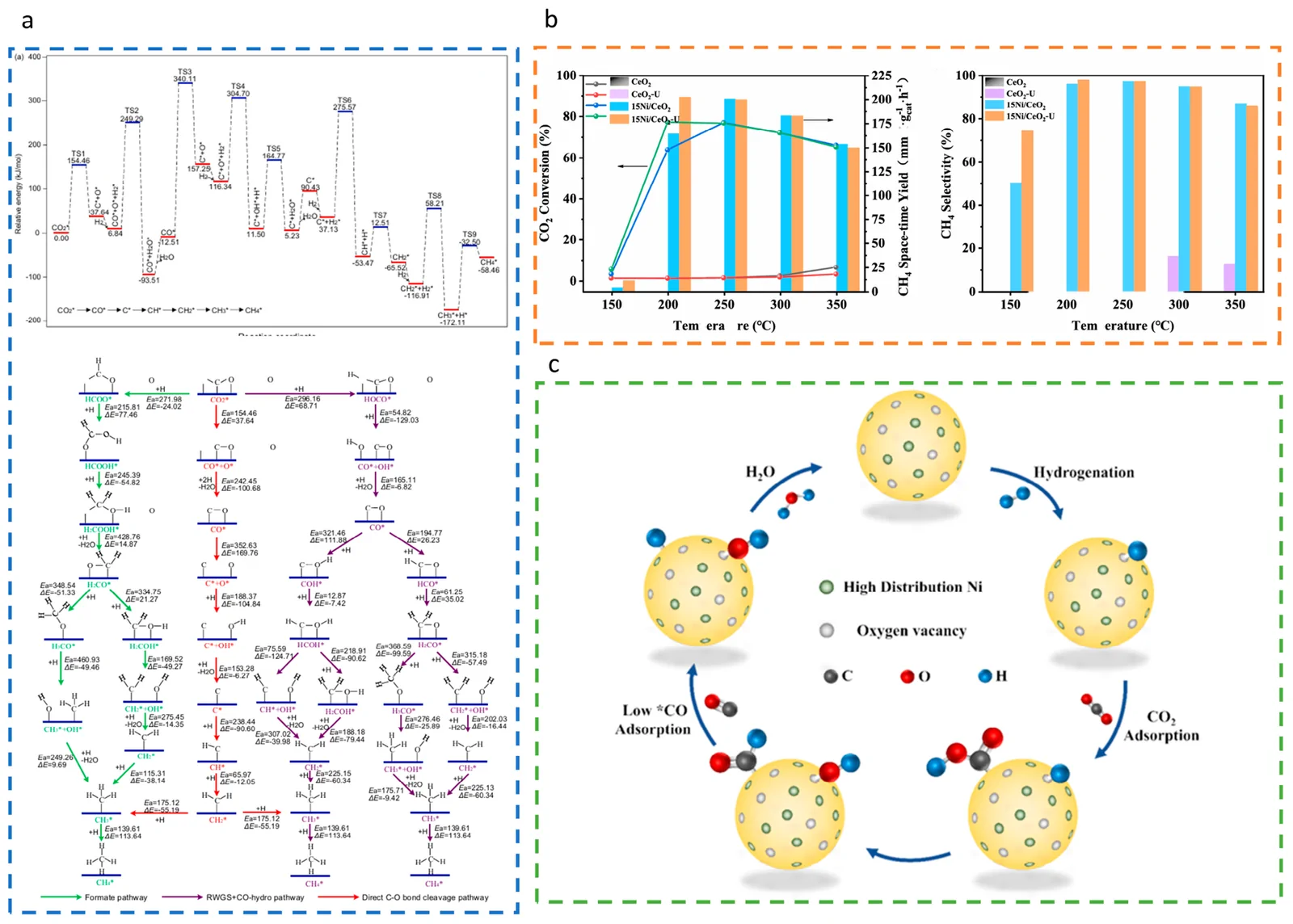
(**a**) The energy diagram of CO_2_ methanation over Ni/CeO_2_ catalyst via the direct C-O bond cleavage pathway. Reaction network of CO_2_ methanation over Ni/CeO_2_ catalyst via the different pathways. Reprinted from Ref. [78], Copyright (2021), with permission from Elsevier. (**b**) CO_2_ conversion, CH_4_ selectivity, and CH_4_ space–time yield. Reprinted from Ref. [81], Copyright (2025), with permission from Elsevier. (**c**) Reaction route of the RWGS reaction over Ni/CeO2-A catalyst. Reprinted from Ref. [82], Copyright (2025), with permission from Elsevier.

The possible pathways can therefore be compared without assigning a universal mechanism to all Ni/CeO_2_ systems. As shown in Figure 4a, direct C–O bond cleavage involves the sequence CO_2_* → CO* → C* → CH* → CH_2_* → CH_3_* → CH_4_*, with calculated barriers of 154.46 kJ mol^−1^ for the initial CO_2_ dissociation and a higher barrier for subsequent CO* cleavage [78]. The same study predicted that the RWGS–CO hydrogenation route is energetically more favourable under the investigated model conditions. Accordingly, Ni/CeO_2_ may exhibit formate-type surface species while still producing CH_4_ predominantly through CO-containing intermediates. The dominant route should therefore be evaluated by combining operando spectroscopy, isotopic tracing, kinetic analysis, and theory rather than inferred from a single detected intermediate.

Ga modification of Ni/CeO_2_ provides a similar selectivity-switching concept. By controlling Ga species distribution to form NiGa_2_O_4_ spinel, the driving force for deep hydrogenation on Ni can be reduced, shifting the Ni-based system from methanation tendency toward low-temperature RWGS CO selectivity [83]. Therefore, the key to product regulation over Ni/CeO_2_ is not simply “enhancing oxygen vacancies,” but creating an appropriate intermediate selectivity among oxygen vacancies, Ni^0^/Niᵟ^+^, and additional metal oxides: HCOO*/CH_x_ paths favor CH_4_, while COOH*/CO* paths favor CO.

Recent Ni-Ce solid-solution studies further demonstrate that Ni/CeO_2_ can also shift from methanation to RWGS by inhibiting CO deep hydrogenation. Fu et al. used a one-pot method to form Ce_1−x_Ni_x_O_2−_ᵧ solid solutions, highly dispersing Ni species in the CeO_2_ lattice; substitution of Ni^2+^ for Ce^4+^ simultaneously creates surface defects and abundant oxygen vacancies, enhancing H_2_ dissociation and inhibiting Ni agglomeration. 5Ni/CeO_2_-A achieved nearly 100% CO selectivity at 300–600 °C, atmospheric pressure, and 40,000 mL g_cat^−1^ h^−1^, with an RWGS rate of 1332 μmol_CO g_Ni^−1^ s^−1^ and almost no deactivation over 50 h [82]. The key to this system is not enhancing Ni’s deep hydrogenation ability, but weakening CO adsorption through highly dispersed Ni, allowing *CO to desorb directly; meanwhile, Ce^3+^-OH participates in CO_2_ adsorption to form bicarbonate/formate intermediates. This starkly contrasts with conventional Ni/CeO_2_ methanation systems, indicating that Ni species size, solid-solution defects, and CO adsorption strength jointly determine the product outlet of Ni/CeO_2_ between CH_4_ and CO.

##### Oxygen Vacancies and CeO_2_ Morphology: Core Descriptors for Low-Temperature Methanation

Oxygen vacancies are the most recurrent structural descriptor in low-temperature CO_2_ methanation over Ni/CeO_2_. They not only enhance CO_2_ adsorption and C-O bond polarization, but also stabilize small Ni particles through enhanced Ni-CeO_2_ interaction, and promote H spillover and oxygenate intermediate hydrogenation. Du et al. used oxygen vacancy-rich CeO_2_ nanoplates to disperse Ni nanoparticles; the resulting Ni/CeO_2_-P achieved over 84% CO_2_ conversion and 100% CH_4_ selectivity at 300 °C, with stable operation for 100 h at 300 °C [84].

Recent oxygen vacancy enrichment strategies have further pushed the low-temperature window to 250 °C or even 200 °C. Yang et al. designed Ni^0^/NiO supported on oxygen vacancy-rich CeO_2_ (Ni/CeO_2_-Ov-R), obtaining about 76% CO_2_ conversion and 100% CH_4_ selectivity at 250 °C, and stable for 80 h at 300 °C [80]. DFT indicated that CeO_2_(110) more readily forms oxygen vacancies; abundant vacancies and exposed Ni^0^ simultaneously promote CO_2_ and H_2_ activation; DRIFTS showed coexistence of CO and formate pathways.

Li et al. employed UiO-66-derived defect engineering to prepare 15Ni/CeO_2_-U, achieving exceptionally outstanding low-temperature activity. In Figure 4b, this catalyst reached 77.1% CO_2_ conversion, 98.7% CH_4_ selectivity, and a CH_4_ STY of 204.0 mmol g_cat^−1^ h^−1^ at 200 °C, considered by the authors as one of the best low-temperature CO_2_ methanation performances for Ni/CeO_2_ catalysts to date [81]. Raman and quasi-in situ XPS revealed richer oxygen vacancies; H_2_-TPR showed that the NiO/CeO_2_ reduction peaks of 15Ni/CeO_2_-U (252 and 357 °C) were lower than those of conventional 15Ni/CeO_2_ (281 and 375 °C), indicating that defect engineering simultaneously improved reducibility and interfacial interaction.

On the other hand, Ni/CeO_2_ can also be steered toward high-selectivity RWGS by regulating the distribution of Ni in the CeO_2_ lattice. In Figure 4c, the reaction route of 5Ni/CeO_2_-A shows that the Ce_1−x_Ni_x_O_2−_ᵧ solid solution highly disperses Ni species and inhibits agglomeration after reduction; Ce-OH sites participate in CO_2_ adsorption to form bicarbonate, which is then converted to formate under surface H*, ultimately yielding CO that desorbs rapidly [82]. In situ DRIFTS observed bicarbonate, formate, and gas-phase CO signals, with no obvious strong *CO adsorption peaks, indicating that weak CO adsorption on this catalyst favors CO desorption and inhibits further hydrogenation to CH_4_. This result contrasts with conventional Ni/CeO_2_ methanation and shows that Ni dispersion and CO adsorption strength, rather than oxygen-vacancy abundance alone, determine whether CH_4_ or CO is obtained.

Plasma-assisted precursor decomposition can also construct highly active Ni-CeO_2_ interfaces. Rui et al. prepared Ni/CeO_2_-P, which achieved a CH_4_ formation rate of 100.3 μmol g_cat^−1^ s^−1^ at 275 °C, with a TOF of 0.49 s^−1^ at 255 °C, about 5 times that of conventional Ni/CeO_2_-C, and maintained >99% CH_4_ selectivity over the entire tested temperature range [50]. In situ XRD/XAS/AP-XPS/DRIFTS indicated that this method promotes the formation of Ni-CeO_2_ interfaces, oxygen vacancies, and a higher Ni^0^/(Ni^0^+Ni^2+^) ratio, facilitating H_2_ dissociation, spillover, and CO_2_ intermediate conversion.

Morphology control not only changes the number of oxygen vacancies but also the Ni-CeO_2_ contact boundary. Gao et al. constructed hollow Ni/CeO_2_ and obtained suitable Ni-CeO_2_ interaction by calcination at 650 °C; hollow Ni/CeO_2_-650 achieved 64.1% CO_2_ conversion at 225 °C with nearly 100% CH_4_ selectivity, and showed essentially no deactivation over 72 h [85]. The O_sur/(O_sur + O_latt) ratio of this system reached 70.4% after calcination at 650 °C, with a CO_2_ desorption amount of 179.0 μmol g_cat^−1^, and H_2_ desorption also increased with enhanced interface.

##### Ni-CeO_2_ Interaction Strength: Balance of Dispersion, Reducibility, and Selectivity

Ni-CeO_2_ interaction needs to be moderate. Too weak leads to Ni sintering and difficult CO_2_ activation; too strong results in Ni being covered by CeO_2_ or forming difficult-to-reduce species, reducing H_2_ dissociation and CO hydrogenation ability. Adhikari et al. re-examined the influence of Ni particle size and found that at 538 K and atmospheric pressure, the apparent activation energy for CH_4_ formation and the CO_2_ conversion TOF based on H_2_ chemisorption were essentially independent of Ni particle size in the 1.3–17 nm range; however, the smallest Ni particles (1.3 nm) showed lower CH_4_ selectivity, indicating that overall CO_2_ conversion and the structural requirements for final CO hydrogenation to CH_4_ are not entirely the same [86].

Activation treatment can also tune the interaction. Lin et al. adjusted the metal-support interaction of Ni/CeO_2_ via low-temperature H_2_ treatment and reduction-oxidation cycling, obtaining 2–3 times higher CO_2_ methanation activity than N_2_- or air-pretreated samples, while maintaining near-complete CH_4_ selectivity and high stability [87]. Direct H_2_-activated samples possessed more surface oxygen vacancies, weak basic sites, and Ni-CeO_2_ interfaces, enhancing H_2_/CO_2_ activation.

Oxygen-vacancy-enriched Ni/CeO_2_ nanostructures provide a low-temperature methanation route that does not rely on a second metal dopant. When Ni nanoparticles are dispersed on oxygen vacancy-rich CeO_2_ nanoplates, CeO_2_ can simultaneously enhance CO_2_ adsorption and interfacial migration of hydrogen species after H_2_ activation, making surface species such as formate and carbonate more readily hydrogenated to CH_4_ [80,84]. UiO-66-derived oxygen vacancy-rich Ni/CeO_2_ also shows that close contact between highly dispersed Ni and defective CeO_2_ can improve low-temperature CO_2_ methanation efficiency and inhibit sintering and carbon deposition to some extent [81]. These results indicate that the enhancement of single-metal Ni/CeO_2_ can be achieved through morphology, precursor, and thermal treatment without necessarily resorting to additional metal promoters.

There is also a non-linear relationship between defect sites and metal-support interaction. Oxygen vacancies favor CO_2_ adsorption and formate formation, but if the Ni-CeO_2_ interaction is too strong, Ni sites may be partially covered or converted to difficult-to-reduce species, limiting H_2_ dissociation and deep hydrogenation of CO intermediates [85,87]. Therefore, the optimization target for Ni/CeO_2_ is not maximizing Ce^3+^ or oxygen vacancy signals, but retaining sufficient exposed Ni^0^ sites, interfacial oxygen vacancies, and moderate basic sites on the reducible CeO_2_ surface. Only when these three are simultaneously present can initial CO_2_ activation, H spillover, and CH_4_ formation be coupled at lower temperatures.

Although MgH_2_ hydrogen storage is not CO_2_ hydrogenation per se, studies on Ni/CeO_2_ for improving MgH_2_ dehydrogenation/hydrogenation kinetics can still serve as supporting evidence for hydrogen activation: the interfacial coupling and oxygen defects of Ni/CeO_2_ promote hydrogen species migration and conversion, indicating that the Ni-CeO_2_-Oᵥ structure has general co-catalytic capability in hydrogen-involved reactions [88].

In summary, the outstanding contribution of Ni/CeO_2_ is its ability to couple CeO_2_’s CO_2_ activation with Ni’s H_2_ dissociation/deep hydrogenation to achieve low-temperature, highly selective CO_2_ methanation. For Ni/CeO_2_ methanation, a Ni^0^–NiO/CeO_2_–Oᵥ interfacial ensemble is a widely proposed working model rather than a universally verified active-site structure. Within this proposed model, Ni^0^ is assigned to H_2_ dissociation, whereas interfacial NiO–CeO_2_ motifs and ceria defects are associated with CO_2_ activation, oxygen migration, and water removal. Compared with Cu/CeO_2_, Ni/CeO_2_ more readily pushes CO_2_ hydrogenation to CH_4_; however, through modulation of intermediates by Mn, In, Ga, etc., selectivity switching between CH_4_ and CO is also possible [51,83,89]. Future design of Ni/CeO_2_ catalysts should focus on four descriptors: (1) the ratio of Ni^0^ to NiO-CeO_2_ interface, avoiding excess of either H_2_ activation or CO_2_ adsorption sites; (2) the concentration of regenerable oxygen vacancies and Ce^3+^ under reaction conditions, especially those on CeO_2_(110), nanoplates, hollow structures, and MOF-derived defect structures; (3) the strength of Ni-CeO_2_ interaction, which should stabilize small Ni particles and interfacial oxygen vacancies while avoiding excessive encapsulation and difficult reduction; (4) H_2_O desorption, sintering resistance, and carbon deposition resistance, which determine long-term stability under high-conversion conditions. Compared with Cu/CeO_2_, Ni/CeO_2_ is more strongly governed by CO* deep hydrogenation and water removal, whereas Cu/CeO_2_ is more dependent on oxygenate stabilization and CO desorption. These design criteria address key technical challenges in industrial CO_2_ methanation for power-to-gas applications [11]. Low-temperature activity enabled by oxygen vacancies reduces preheating energy demand and improves compatibility with fluctuating renewable hydrogen supplies. Sintering and carbon deposition resistance are critical for long-term operation under the highly exothermic conditions of methanation reactors, where hotspots commonly cause catalyst degradation [10]. Current state-of-the-art Ni/CeO_2_ catalysts achieve high conversion at 200–300 °C in laboratory tests, but performance under realistic feeds containing sulfur impurities, higher hydrocarbons, and thermal cycling remains insufficiently characterized. Sulfur impurities at ppm levels are known to significantly reduce the service life of conventional Ni-based methanation catalysts, and ceria-based supports have been proposed as a strategy to moderate sulfur tolerance [30]. Scaling to industrial reactors will also require structured catalyst forms to manage heat transfer and pressure drop [10].

#### 3.1.3. Other Metal-Modified Ceria

##### Other Metal/CeO_2_ Interfaces: From “Single Support” to “Selectivity-Programmable” Synergy Platforms

In the CO/CO_2_ hydrogenation over CeO_2_, Cu and Ni represent the two typical endpoints of methanol/RWGS regulation and methanation deep hydrogenation, respectively. Au, Pd, Pt, Rh, Ru, Co, Ir, and doped CeO_2_ systems further demonstrate that the true value of CeO_2_ is not as an inert support, but as a “reaction pathway selector” that tunes oxygen vacancies, Ce^3+^/Ce^4+^ cycling, interfacial oxygen chemical potential, and metal valence/nuclearity. For metals with weak C-O deep-hydrogenation ability such as Au and Pd, CeO_2_ more often acts as a platform for CO_2_ polarization, formate/carbonate intermediate stabilization, and selective hydrogenation interfaces. For strongly hydrogenating metals such as Co, Ru, and Ir, CeO_2_ controls CH_4_/CO splitting through morphology, defects, and SMSI to limit metal sintering. For Rh or Pd single-atom/dimer systems, the oxygen vacancies and metal-oxygen coordination on CeO_2_ can further participate in C-C coupling, extending CO_2_ hydrogenation from C_1_ products to C_2_ oxygenates such as ethanol [52,90,91,92,93,94,95].

Thus, different metals do not simply replace Cu or Ni, but reshape the outlet of the CO_2_ hydrogenation network by modifying H_2_ dissociation, CO* binding strength, interfacial oxygen vacancy stability, and metal nuclearity. This is prominently illustrated by the high-selectivity ethanol production over Pd_2_/CeO_2_, the single-atom ethanol pathway over Rh_1_/CeTiO_x_, the high-selectivity methanation over Ir/GDC nanorods, and the mid-temperature CO_2_ deoxygenation over In-doped CeO_2_ [91,92,95,96].

##### Au and Pd: Weak Deep-Hydrogenation Metals Promote Selective CO_2_ Activation and Oxygenate Conversion

The selective hydrogenation characteristics of Au/CeO_2_ are also illustrated by aldehyde hydrogenation under syngas conditions. CeO_2_-supported Au nanoparticles maintain selective aldehyde hydrogenation in the presence of CO/H_2_, where CO acts more as a reversible site blocker, suppressing side reactions without significantly reducing the target hydrogenation rate; the Au particle size changed from an average of 2.5 nm in fresh samples to 2.4 nm after the third cycle, indicating good stabilization of Au nanoparticles by CeO_2_ [97].

**Figure 5:**
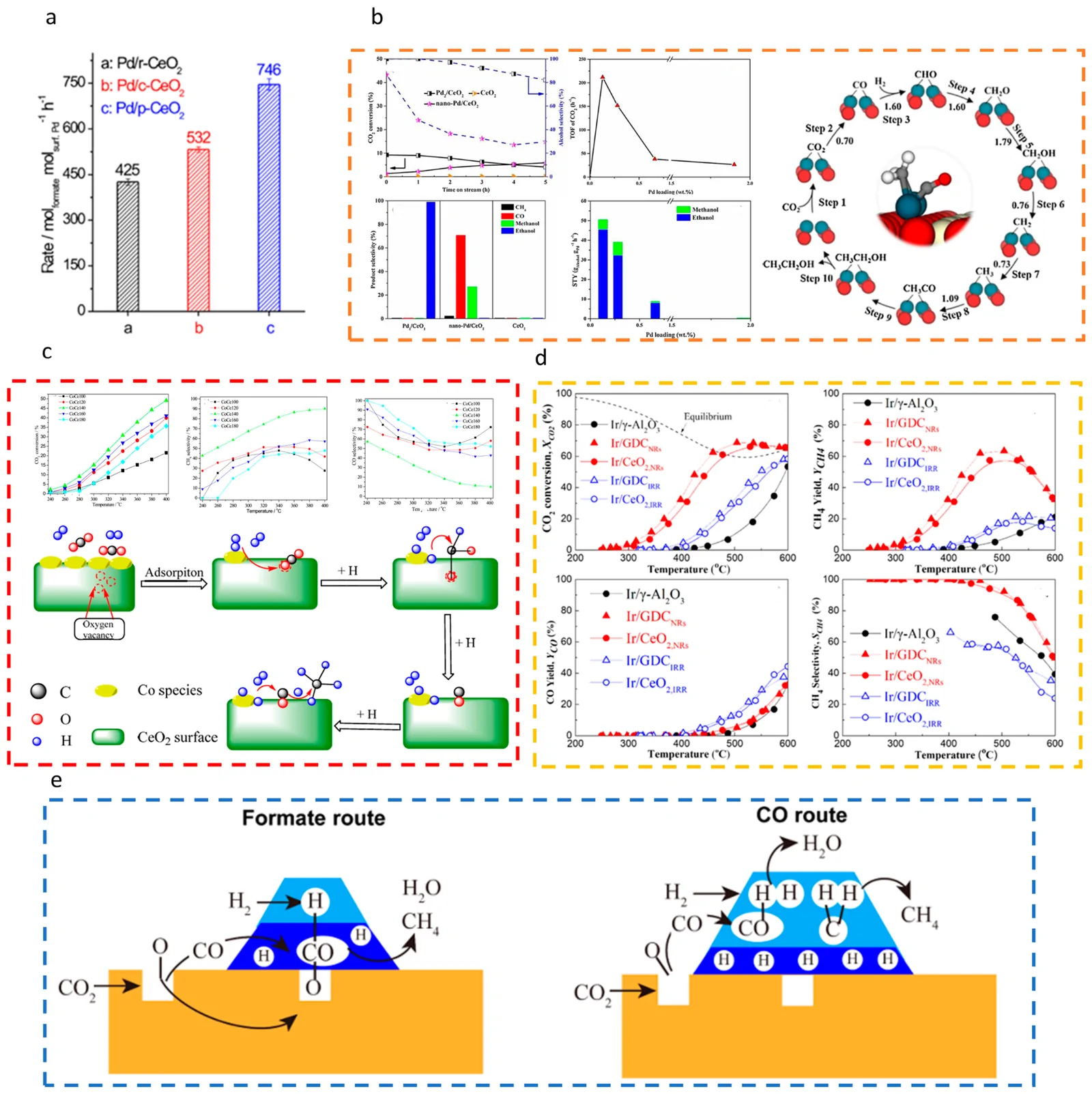
(**a**) Formate production normalized to surface Pd atoms. Reprinted from Ref. [98], Copyright (2021), with permission from Elsevier. (**b**) CO_2_ hydrogenation over CeO_2_, Pd/CeO_2_, and Pd_2_/CeO_2_, including calculated barriers for ethanol formation over Pd_2_/CeO_2_(110). Reprinted from Ref. [92], Copyright (2021), with permission from Elsevier. (**c**) CO_2_ conversion and CH_4_/CO selectivity over Co/CeO_2_−δ, together with the proposed reaction pathway. Reprinted from Ref. [52], Copyright (2020), with permission from Elsevier. (**d**) CO_2_ hydrogenation light-off curves for Ir nanoparticles supported on ceria-based materials and γ-Al_2_O_3_. Reprinted from Ref. [91], under CC BY 4.0 license. (**e**) Proposed formate and CO pathways for CO_2_ methanation. Reprinted from Ref. [99], Copyright (2026), with permission from Elsevier.

Pd/CeO_2_ exhibits even richer selective oxygenate pathways. In Figure 5a, Fan et al. compared Pd/CeO_2_ nanorods, nanocubes, and polyhedra for CO_2_ hydrogenation to formate. They found that the oxygen vacancy concentration follows rods > cubes > polyhedra, but the intrinsic activity does not simply increase with vacancies; Pd/p-CeO_2_ gave the highest TOF of 746 h^−1^, higher than 532 h^−1^ for Pd/c-CeO_2_ and 425 h^−1^ for Pd/r-CeO_2_ [98]. This indicates that too strong a Pd-CeO_2_ interaction makes Pd positively charged, weakening H_2_ activation and formate formation, whereas a moderate metallic Pd-CeO_2_ interface is more favorable for converting bidentate carbonate/bicarbonate to formate.

An even more striking example is CeO_2_-supported Pd dimers for CO_2_ hydrogenation to ethanol. In Figure 5b, Lou et al. reported that Pd_2_/CeO_2_ achieves 99.2% ethanol selectivity, an ethanol STY of 45.6 g_ethanol g_Pd^−1^ h^−1^, and 9.2% CO_2_ conversion, significantly higher than 5.3% for nano-Pd/CeO_2_; its TOF was 211.7 h^−1^, also much higher than 26.5 h^−1^ for nano-Pd/CeO_2_. DFT calculations for the Pd_2_O_4_ model indicate a feasible route involving CO_2_ dissociation to CO*, strong CO* binding, and subsequent CO* + CH_3_* coupling. Together with the reported structural and catalytic data, these results are consistent with metal nuclearity affecting the accessibility of C–C coupling on Pd/CeO_2_, although the operative dimer configuration and individual elementary steps require further validation under reaction conditions [92].

PdZn/CeO_2_ further illustrates the influence of Pd alloying and CeO_2_ defect regulation on methanol selectivity. Malik et al. reported that Ca-doped PdZn/CeO_2_ achieves nearly 100% methanol selectivity and 7.7% CO_2_ conversion at 220 °C, 30 bar, and GHSV = 2400 mL g^−1^ h^−1^, with PdZn nanoparticles of about 3–6 nm [100]. Jiang et al. systematically compared CeO_2_ rods, cubes, octahedra, and polyhedra for Pd/CeO_2_ methanol synthesis, pointing out that 2Pd/CeO_2_-R, exposing the (110) facet rich in oxygen mobility, has the highest oxygen vacancy concentration and CO_2_ hydrogenation activity; however, the TOF normalized per single oxygen vacancy on 2Pd/CeO_2_-O was about 15 times that of 2Pd/CeO_2_-R, indicating that “easy formation of vacancies” and “reactivity of individual vacancies” need to be balanced. DFT further showed that CH_3_OH is more likely generated via the formate pathway, where HCOO* hydrogenation to HCOOH* is the rate-limiting step [43]. Cao et al., through defect engineering, achieved size control of Pd nanoparticles on CeO_2_ and found that larger Pd nanoparticles, with higher electron density and stronger H_2_ dissociation/H spillover, generate more oxygen vacancies for CO_2_ activation during reaction, thus exhibiting higher intrinsic activity in CO_2_-to-CO hydrogenation [101].

##### Co, Ru, and Ir: Methanation, SMSI, and Dynamic Interface Regulation in Strong Hydrogenation Metals

Co/CeO_2_, similar to Ni/CeO_2_, has a strong tendency for CO_2_ deep hydrogenation, but its selectivity is more susceptible to CeO_2_ morphology, oxygen vacancies, and Co-CeO_2_ interface reconstruction. In Figure 5c, Xie et al. studied the morphology effect of Co/CeO_2−_δ and found that CeO_2_ nanorods exposing (110)/(100) facets promote Co ion incorporation into the CeO_2_ lattice to form Ce-O-Co solid-solution structures, generating more Co^0^, oxygen vacancies, and Ce^3+^-Co^0^ structures; the optimal CoCe140 catalyst exhibited outstanding performance in the 240–400 °C range, with CO_2_ conversion reaching 48.7%, CH_4_ selectivity 91.7%, and CO selectivity 8.3% [52].

The Co-CeO_2_ interface is not static. Gao et al. constructed inverse CeO_x_/Co catalysts and demonstrated that CeO_2−x_ clusters strongly stabilize Co nanoparticles, suppressing Co sintering during 500 °C H_2_ reduction and CO_2_ hydrogenation; the inverse catalyst with 20 mol% Ce showed a methanation rate one order of magnitude higher than Co catalysts without CeO_2_. In this system, CeO_2−x_ clusters not only stabilize metallic Co, but also activate CO_2_ to carbonyl CO* intermediates that transfer to Co sites for further hydrogenation [90]. Chen et al. further proposed that “local oxygen chemical potential” determines the in situ reconstruction of the Co-CeO_2_ interface in CO_2_ hydrogenation: the local μO in the reaction atmosphere controls the reduction/oxidation state of Co species and the formation of interfacial active sites, thereby influencing the CO_2_ hydrogenation pathway [102].

The CeO_2_ particle size and exposed surface are equally important for CO/CH_4_ splitting over Co/CeO_2_. Oh et al. used 5 wt% Co/CeO_2_ and varied the CeO_2_ calcination temperature; when CeO_2_ was calcined at 1000 °C, the CO selectivity increased from 24 ± 2% to 49 ± 1%. They attributed this to the improved reducibility of CeO_2_, enhanced CO adsorption, and weakened H adsorption on Co nanoparticles, thereby inhibiting further CO hydrogenation to CH_4_ [103]. Deng et al., using in situ XAFS/XRD/DRIFTS and environmental TEM, directly visualized the size-dependent dynamic behavior of CoO_x_ nanoparticles supported on CeO_2−_δ(100) under CO_2_/H_2_ conditions, proving that strong CoO_x_-CeO_2_ interaction stabilizes the CeO_2_ (100) facet and induces oriented CoO_x_ nanoparticles [104]. These two works together show that selectivity control over Co/CeO_2_ is essentially the dynamic equilibrium among CoO_x_/Co, Ce^3+^/Ce^4+^, and interfacial oxygen vacancies under reaction conditions, rather than a simple extrapolation of fresh catalyst structures.

Ir/CeO_2_ or Ir/GDC systems are noble-metal examples of highly selective methanation. In Figure 5d, Nikolaraki et al. found that morphology-controlled CeO_2_ and Gd-doped CeO_2_ (GDC) nanorods significantly enhance Ir-catalyzed CO_2_ methanation; the TOF of Ir/GDC nanorods at 380 °C was 26-fold and 10-fold higher than Ir/Al_2_O_3_ and irregular Ir/CeO_2_ particles, respectively. The optimal Ir/GDC nanorods achieved about 70% CO_2_ conversion, about 95% CH_4_ selectivity, and about 64% CH_4_ yield at about 500 °C, maintaining near-100% CH_4_ selectivity in the effective temperature range. DRIFTS indicated that the reaction proceeds via formate conversion to Ir-adsorbed CO species and then to CH_4_; the Ir-Ce^3+^ interface lowered the apparent activation energy by about 37 kJ mol^−1^ [91].

Ru/CeO_2_ further demonstrates that the activity of noble-metal-ceria interfaces is governed not only by the metal type, but also by Ru size, Ruᵟ^+^/Ru^0^ ratio, and CeO_2_ morphology. In Figure 5e, Ru/CeO_2_-rod achieved about 70% CO_2_ conversion and 98% CH_4_ selectivity at 300 °C, while Ru/CeO_2_-cube gave about 50% conversion and 96% CH_4_ selectivity; under differential conditions, the specific reaction rate of Ru/CeO_2_-rod was 8.52 mmol g_Ru^−1^ s^−1^, higher than 6.21 mmol g_Ru^−1^ s^−1^ for Ru/CeO_2_-cube [99]. Structural characterization showed that Ru on rod-shaped CeO_2_ exists as clusters of about 1.2 nm with 2–3 atomic layers, and has higher Ce^3+^ and oxygen vacancy concentrations; the corresponding Ruᵟ^+^/Ru^0^ ratio was also higher than that of cube samples. Thus, the Ru^0^-Ruᵟ^+^-Oᵥ-Ce interface can be understood as a synergistic site: oxygen vacancies activate CO_2_, Ru sites dissociate H_2_ and hydrogenate carbonyl/formate intermediates, echoing the bifunctional picture of “oxide activates CO_2_, metal activates H_2_” in Ni/CeO_2_.

Ru/CeO_2_ is another highly active methanation system, but its interface strength requires delicate balance. Wang et al. investigated different Ru loadings and found that 1% Ru/CeO_2_ performs best in low-temperature CO_2_ methanation; oxygen vacancy concentration shows a volcanic trend with Ru loading, and Ru-CeO_2_ interaction influences CO_2_ activation and H_2_ dissociation [94]. Single-atom Ru immobilized on hierarchical porous CeO_2_ can increase oxygen vacancies for CO_2_-to-formic acid, indicating that the dispersion state of Ru from particles to single atoms changes the product outlet [105]. Lv et al. proposed a mechanocatalytic approach that enhances Ru-CeO_2_ interaction and Ru redispersion at low temperatures, significantly accelerating CO_2_ methanation below 160 °C in a vibratory reactor; other studies tuned Ru-CeO_2_ interfacial structure via CeO_2_ nanorods/cubes, showing that support morphology determines oxygen vacancy density, Ru cluster dispersion, and methanation activity [99,106].

Notably, SMSI does not necessarily bring higher methanation activity. Dang et al. compared Ru(N)/CeO_2_ and Ru(Cl)/CeO_2_ and found that N-induced SMSI led to more oxygen vacancies and CeO_2−x_ encapsulation layers on Ru(N)/CeO_2_, but the exposed Ru atoms decreased, limiting H_2_ activation and H spillover, thus lowering activity and shifting selectivity toward CO; Ru(Cl)/CeO_2_, with more exposed Ru atoms, followed an H_2_-associative pathway and exhibited higher methanation activity and CH_4_ selectivity. Kinetically, the apparent activation energy of Ru(N)/CeO_2_ was 65.3 ± 1.5 kJ mol^−1^, while that of Ru(Cl)/CeO_2_ was 96.2 ± 1.5 kJ mol^−1^, but the former did not show higher overall activity due to limited H_2_ activation; its H_2_ reaction order was close to zero, whereas Ru(Cl)/CeO_2_ had positive orders for both H_2_ and CO_2_ [107].

Additionally, Co as a modifier or promoter in Ni/CeO_2_ or reforming systems can be used to discuss coking resistance and metal dispersion in CeO_2_ composite interfaces. Babakouhi et al. introduced Cr/Mn/Co into 10%Ni/Al_2_O_3_-10%CeO_2_ and found that Cr modification achieved 70.5% CH_4_ and 69.1% CO_2_ conversion in dry reforming with reduced coking [108].

##### Rh, Pd Dimers, and Ti Doping: From C_1_ Hydrogenation to C-C Coupling and Ethanol Synthesis

Rh/CeO_2_ occupies a special position in CO/CO_2_ hydrogenation: Rh possesses both strong CO dissociation and hydrogenation ability, and can promote C-C coupling on suitable CeO_2_ interfaces to produce ethanol. Hong et al., by selecting CeO_2_ crystal facets to tune Rh-CeO_2_ interaction, found that Rh/CeO_2_ nanorods performed best in CO_2_ hydrogenation, with ethanol selectivity of 20.9%, CO_2_ conversion of 11.2%, and ethanol production rate of 69.2 mmol g_Rh^−1^ h^−1^; the different exposed facets of CeO_2_ nanorods, nanocubes, and nanosheets led to differences in Rh dispersion, valence, and interfacial oxygen vacancies [93].

The selectivity of Rh/CeO_2_ is also limited by the combined effects of Rh particle size and CeO_2_ morphology. Liao et al., by decoupling CeO_2_ morphology and Rh particle size in interfacial catalysis, correlated a higher density of interfacial oxygen vacancies with higher CO_2_-hydrogenation activity, whereas changes in Rh nuclearity and morphology were associated with product selectivity. These comparative trends identify relevant structural descriptors, but do not by themselves establish a unique active site; smaller Rh particles, with weakened metallicity, can suppress Sabatier methanation and promote RWGS to CO [44]. Therefore, single-metal Rh/CeO_2_ serves as a basic model for understanding noble-metal-CeO_2_ interface selectivity: CeO_2_ determines the CO_2_ activation boundary, while the nuclearity and electronic state of Rh govern the accessibility of CO, CH_4_, or oxygenate pathways.

The dynamic evolution of Rh single atoms on CeO_2_ is also worth noting. Wu et al. studied CeO_2_ trapping of Rh single atoms for CO hydrogenation and found that among 0.2–4 wt% Rh/CeO_2_, the highest atomic dispersion of about 3 wt% could be achieved; Rh single atoms were stable in H_2_ up to 350 °C, but formed Rh nanoclusters in CO at 200 °C. In syngas conversion, Rh single atoms on 0.5 wt% Rh/CeO_2_ slowly agglomerated, and after about 10 h the Rh_iso/Rh_NC ratio stabilized at about 1:2.5 [109]. This reminds us that “single-atom catalysts” under CO/CO_2_ hydrogenation conditions are often dynamic coexistence systems of single atoms and clusters, and the true active sites need to be determined by in situ/operando characterization.

Pd/CeO_2_ further shows that metal nuclearity can change C-C coupling probability without introducing a second metal. CeO_2_-supported Pd dimers can tune the proximity of CO* and CH_3_* and promote ethanol formation in CO_2_ hydrogenation; compared with conventional Pd nanoparticles, dimer sites are more favorable for limiting excessive hydrogenation and enhancing C_2_ oxygenate selectivity [92]. This indicates that selectivity control over single-metal/CeO_2_ comes not only from the metal type, but also from nuclearity transitions among single atoms, dimers, clusters, and nanoparticles.

The common lesson from Pd/CeO_2_, Rh/CeO_2_, and Ru/CeO_2_ is that noble-metal sites must maintain moderate contact with CeO_2_ defects. If the metal is overly dispersed as isolated single atoms that are unstable during reaction, they may re-agglomerate under CO or H_2_ conditions; if particles are too large, the interface fraction decreases, weakening CO_2_ activation and oxygenate stabilization [98,101,109]. Therefore, this section treats noble-metal/CeO_2_ as a single-metal system controlled by the triad of “nuclearity–interface–defects,” rather than simply ranking Pd, Rh, and Ru by intrinsic activity.

##### Pt, Zn, and In/Zr/Gd/Sm Doping: Supporting Evidence for Interfacial Coordination and Oxygen Vacancy Engineering

Pt_1_/CeO_2_, although mainly reported for CO oxidation, has methodological implications for CO/CO_2_ hydrogenation through its “facet-dependent Pt-O coordination.” Yan et al., by directing the exposed facets of CeO_2_, tuned the diversity of Pt_1_-O coordination, demonstrating that the coordination environment of single-atom Pt can be oriented by CeO_2_ facets; Pt_1_/CeO_2_-IMP achieved complete CO conversion at 175 °C with an initial TOF of about 25 s^−1^ [110]. For CO_2_ hydrogenation, this suggests that the activity of single-atom metal/CeO_2_ cannot be described merely by “metal single atom,” but must specify the Pt-O-M or Pt-O-Ce coordination number, facet, and oxygen vacancy environment.

Pt_1_/CeO_2_ and other single-atom/CeO_2_ models also indicate that interfacial coordination is more explanatory than the metal name itself for activity differences. The Pt-O-Ce coordination number, exposed facets, and neighboring oxygen vacancies of single-atom Pt alter the electronic structure of adsorbed species; similarly, dynamic reconstruction of CoO_x_, Pd, or Rh on CeO_2_ under reaction atmospheres changes the actual active sites [90,104,110]. Therefore, the description of single-metal/CeO_2_ should move from “metal X supported on CeO_2_” to “metal sites of nuclearity Y working on specific CeO_2_ facets and defect environments.”

In-doped CeO_2_ provides evidence for oxygen vacancy-mediated CO_2_ deoxygenation without noble metals. Chiang et al. compared Zr, Gd, Sm, and In doping in CeO_2_ and found that only In-doped CeO_2_ showed significant activity in mid-temperature CO_2_-to-CO deoxygenation up to 700 °C; In_0_._5_Ce_0_._5_Oᵧ after H_2_-TPR had a reduction onset temperature as low as 400 °C and exhibited stable cyclic performance. Structural characterization showed that In exsolves from the fluorite structure during TPR, and the exsolved In^0^ is oxidized during CO_2_ deoxygenation, while Ce also participates in the redox cycle; in contrast, pure In_2_O_3_ has a higher onset temperature, lower capacity per mass, and poorer stability, while CeO_2_ itself is inactive [96]. This indicates that the synergy of doped CeO_2_ is not limited to “creating oxygen vacancies,” but includes the coupled redox cycle between reversible metal/oxide exsolution and the CeO_2_ fluorite framework.

Table 2, Table 3 and Table 5 summarize catalytic data as reported in the original studies. Temperature and pressure are presented in °C and MPa, respectively, whereas space velocity is reported in the original basis because some studies use mass-based flow rates and others use volumetric GHSV. Conversion and selectivity are identified by the reacting carbon source and target product. TOF, STY, yield, and product-formation rates are retained with their original normalization bases because values normalized to catalyst mass, active-metal mass, exposed metal sites, or nominal metal loading are not interchangeable. Accordingly, these tables provide a structured map of reported operating windows, product tendencies, and stability tests, rather than a universal activity ranking. Comparisons should be made primarily within the same target reaction and under reasonably similar feed composition, pressure, temperature, space velocity, and metric definition.

**Table 2:** Reported catalytic performance of single-metal-modified CeO_2_ catalysts, organized by target reaction.

Catalyst	Product	Temperature (°C)	Pressure (MPa)	GHSV/Space Velocity	Conversion (%)	Selectivity (%)	TOF/STY (Type and Units)	Stability Test Duration (h)	Ref.
1 wt% Cu/CeO_2_	CO	600 °C	0.1	360,000 mL·g_cat^−1^·h^−1^	70 (CO_2_)	~100 (CO)	STY: 1640 mmol_CO·g^−1^·h^−1^	20 h	[67]
CeO_2_@Cu-A	CO	400 °C	0.1	360,000 mL·g_cat^−1^·h^−1^	51.3 (CO_2_)	~100 (CO)	STY: 1640 mmol_CO·g^−1^·h^−1^	15 h	[47]
Cu/CeO_2_-400	CO	400 °C	0.1	360,000 mL·g_cat^−1^·h^−1^	-	100 (CO)	STY: 1230 mmol_CO·g^−1^·h^−1^	-	[48]
Cu@CeNF	CO	350 °C	0.1	48,000 mL·g_cat^−1^·h^−1^	28.9 (CO_2_)	>99 (CO)	STY: 210.8 mmolCO·g^−1^·h^−1^	>228 h	[59]
Cu/CeO_2_ (Ce/(Ce+Cu) = 0.67)	CH_3_OH	220 °C	1.5	18,000 mL·gcat^−1^·h^−1^	<10 (CO)	>97 (CH_3_OH)	TOF: 43.2 h^−1^	~100	[58]
Cu/CeO_2_	CH_3_OH	280 °C	3.0	10,000 mL·gcat^−1^·h^−1^	6.8 (CO_2_)	>50 (CH_3_OH)	-	-	[61]
Ni/CeO_2_-NR	CO/CH_4_	350 °C	0.1	30,000 mL·gcat^−1^·h^−1^	90 (CO_2_)	~100 (CH_4_)	Rate: r_CO_2_ ~2.88 mol·g_Ni^−1^·h^−1^	-	[51]
Ni/CeO_2_	CH_4_	400 °C	~0.1	30,000 mL·gcat^−1^·h^−1^	45 (CO_2_)	99 (CH_4_)	-	24 h	[111]
15Ni/CeO_2_-U	CH_4_	200 °C	0.1	30,000 mL·gcat^−1^·h^−1^	77.1 (CO_2_)	98.7 (CH_4_)	TOF: 182.8 h^−1^	100 h	[81]
16 wt% Ni/CeO_2_	CH_4_	265 °C	0.1	100 mL/min total	5.8 (CO_2_)	~90 (CH_4_)	TOF: 72 h^−1^	-	[86]
Ni/CeO_2_-650	CH_4_	225 °C	0.1	36,000 mL·gcat^−1^·h^−1^	64.1 (CO_2_)	~100 (CH_4_)	-	72 h	[85]
Ni-US (I)	CH_4_	375 °C	0.1	12,000 h^−1^	70 (CO_2_)	~100 (CH_4_)	-	-	[74]
Ni/(0.06AB)CeO_2_	CH_4_	240 °C	0.1	36,000 mL·gcat^−1^·h^−1^	67.5 (CO_2_)	99.6 (CH_4_)	TOF: 972 h^−1^	100 h	[79]
Ni/CeO_2_-Ov-R	CH_4_	250 °C	0.1	20,000 mL·gcat^−1^·h^−1^	76 (CO_2_)	~100 (CH_4_)	-	80 h	[80]
Ni/CeO_2_-P	CH_4_	300 °C	0.1	6000 mL·gcat^−1^·h^−1^	84 (CO_2_)	~100 (CH_4_)	TOF: 172.1 h^−1^	100 h	[84]
Ni/CeO_2_-SGM	CH_4_	250 °C	0.1	40,000 mL·gcat^−1^·h^−1^	80.5 (CO_2_)	95.8 (CH_4_)	Rate: Activity = 1.30 mol/g_Ni/h	106 h	[73]
Co/CeO_2_-NR	CO/CH_4_	350 °C	0.1	30,000 mL·gcat^−1^·h^−1^	~46 (CO_2_)	~90 (CH_4_)	Rate: r_CO_2_ ~ 1.602 mol·g_Co^−1^·h^−1^	-	[51]
20Ce80Co	CH_4_	200 °C	0.1	-	-	>90 (CH_4_)	Metric: 7.20 mol·(mol Co)^−1^·h^−1^	20 h	[102]
CoCe140	CH_4_	400 °C	~0.1	60,000 mL·gcat^−1^·h^−1^	48.7 (CO_2_)	91.7 (CH_4_)	-	7.3 h	[108]
17FeCe	CO	600 °C	0.3	60,000 mL·gcat^−1^·h^−1^	56 (CO_2_)	~100 (CO)	-	-	[68]
Pd/CeO_2_-0.1	CO	275 °C	~0.1	-	-	100 (CO)	TOF: 506 h^−1^	-	[101]
1% Ru/CeO_2_	CH_4_	300 °C	~0.1	-	86 (CO_2_)	~100 (CH_4_)	-	30 h	[94]
Ir/CeO_2_-NR	CH_4_	500 °C	~0.1	-	70 (CO_2_)	~95 (CH_4_)	-	12 h	[91]
Ru(Cl)/CeO_2_	CH_4_	350 °C	~0.1	-	>10 (CO_2_)	100 (CH_4_)	-	-	[107]
Ru-CeO_2_-grinding	CH_4_	160 °C	~0.1	-	92 (CO_2_)	100 (CH_4_)	-	12 h	[106]
Ru/CeO_2_-rod	CH_4_	300 °C	~0.1	60,000 mL·gcat^−1^·h^−1^	70 (CO_2_)	98 (CH_4_)	STY: 30,672 mmol·g_Ru^−1^·h^−1^	50 h	[99]
2Pd/CeO_2_-R	CH_3_OH	240 °C	3.0	2000 mL·gcat^−1^·h^−1^	-	47.7 (CH_3_OH)	TOF: (Pd): 79.8 h^−1^	100 h	[43]
1Rh/CeO_2_	C_2_H_5_OH	260 °C	2.0	10,000 mL·gcat^−1^·h^−1^	-	26.2 (C_2_H_5_OH)	STY: 308.9 mmol·g_Rh^−1^·h^−1^	>20 h	[109]
Pd_2_/CeO_2_	C_2_H_5_OH	240 °C	3.0	3000 mL·gcat^−1^·h^−1^	9.2 (CO_2_)	99.2 (C_2_H_5_OH)	STY: 989.8 mmol_ethanol·g_Pd^−1^·h^−1^	-	[92]
Rh/CeO_2_-r	C_2_H_5_OH	250 °C	3.0	6000 mL·gcat^−1^·h^−1^	11.2 (CO_2_)	20.9 (C_2_H_5_OH)	STY: 69.2 mmol·(gRh)^−1^·h^−1^	50 h	[93]

**Note:** Values are reproduced from the original studies with unit harmonization where unambiguous. Temperature is given in °C and pressure in MPa. TOF values originally reported in s^−1^ were converted to h^−1^. Rate and STY values retain the original catalyst or metal-mass normalization basis. GHSV or space velocity is reported according to the normalization basis available in each source. A dash (-) indicates that the corresponding value was not reported. The values should not be directly ranked across different reaction conditions and normalization bases. As summarized in Table 2, single-metal-modified CeO_2_ catalysts exhibit different product tendencies across the reported reaction classes. Within the CO_2_-to-CO studies, Cu/CeO_2_ commonly shows high CO selectivity under high-temperature and low-pressure RWGS conditions. Ni-, Co-, Ru-, and Ir-containing catalysts are more frequently associated with CO_2_ methanation, whereas Pd- and Rh-containing systems extend the reported product range toward methanol and C_2_ oxygenates. These observations describe product-selectivity tendencies within the assembled literature rather than a direct ranking of intrinsic catalytic activity, because the reported operating conditions and performance-normalization methods are not uniform.

This comparison shows that “optimal” must be judged together with target product, reaction conditions, selectivity, space-time yield, and stability. For the purpose of this review, this table serves as a horizontal index for product-oriented design rather than a simple activity ranking. These cases together illustrate that “single-metal” in single-metal/CeO_2_ does not imply that the active site is simple. Metal nuclearity, metal-oxygen coordination, CeO_2_ facets, oxygen vacancy location, and reaction-induced reconstruction often simultaneously determine selectivity. Therefore, defining a catalyst solely by the metal element name is often insufficient to explain its reaction differences; more critical is describing its interfacial configuration under reaction conditions.

### 3.2. Bimetal-Modified Ceria

The value of bimetallic/CeO_2_ systems lies in moving selectivity control from empirical promotion to definable interfacial combinations. The second metal can form alloys to modify the electronic structure of the main metal, anchor as single atoms at CeO_2_ defect sites, or alter oxygen vacancy formation energy and surface basicity through heterovalent doping. PdZn and CuZn systems emphasize the coupling of CO_2_ activation, H_2_ supply, and methoxy hydrogenation in the methanol pathway; NiY and La-M-CeO_2_ systems highlight the balance of Ni dispersion, basic sites, and oxygen mobility in low-temperature methanation; Pt-Co/Pt-Ni dual-single-atom systems provide selectivity-splitting evidence closer to model catalysts. It should be noted that bimetallic effects are not always positive; overly strong interaction, excessive encapsulation, or excess CO_2_ adsorption can reduce effective H_2_ activation or block key intermediate conversions [46,112,113,114,115,116]. Therefore, the most valuable evidence for bimetallic systems is not “performance improvement after adding a second metal,” but rather the ability to specify which rate- or selectivity-determining step the second metal has altered. For example, does it enhance H_2_ dissociation, weaken excessive CO* adsorption, stabilize methoxy intermediates, increase regenerable interfacial oxygen vacancies, or change water desorption and carbonate coverage? Only by disentangling these functions can bimetallic/CeO_2_ move from empirical formulations to designable site engineering.

#### 3.2.1. Core Significance of Bimetallic/CeO_2_: Coupling “Metal Sites” and “Oxygen Vacancy Sites” into a Tunable Reaction Network

Compared with single-metal/CeO_2_, the advantage of bimetallic or bimetallic-doped systems is not merely adding one more active component, but redistributing the three key steps in CO_2_ hydrogenation, CO_2_ adsorption/polarization, H_2_ dissociation/spillover, and C-O/C-H bond transformation, through alloying, heterometal electron transfer, promoter-induced oxygen vacancies, and interfacial geometric constraints. For methanol synthesis, PdZn, PdCu, CuZn, and W/Ca/Zn promoters mainly improve CH_3_OH selectivity by stabilizing formate/methoxy intermediates, suppressing RWGS, and tuning the electronic structure of Cu/Pd. For methanation, NiFe, Ni-Y, or Ni/La-M-CeO_2_ advance CH_4_ formation through Ni’s H_2_ dissociation, second-metal regulation of CO_2_/CO intermediates, and CeO_2_ oxygen vacancies. For RWGS, NiFe or La-doped CeO_2_ can direct interfacial redox cycles and alloy stability toward high-temperature CO production. For C_2_ oxygenates, Ti-doped CeO_2_ stabilizing Rh single atoms or dual-single-atom Pt-Co/Pt-Ni further demonstrate atomically precise multi-metal site control over pathway selection [46,95,112,113,116,117,118].

Therefore, the most striking feature of bimetallic/CeO_2_ systems is not “adding a metal improves activity,” but “the second metal changes the division of labor between CeO_2_ oxygen vacancies and the main metal sites”: it can shift CO_2_ activation from the MvK-RWGS to the formate pathway, and can also separate H_2_ dissociation, CO* hydrogenation, and sintering resistance on different sites. This is clearly illustrated in W-Cu/CeO_2_, Ce-CuZn, PdZn-Ca/CeO_2_, and CeO_2_-confined NiFe [46,112,116,118].

#### 3.2.2. Methanol Synthesis: Formate Pathway Selection in PdZn, PdCu, CuNi, and CuZn-CeO_2_ Interfaces

**Figure 6:**
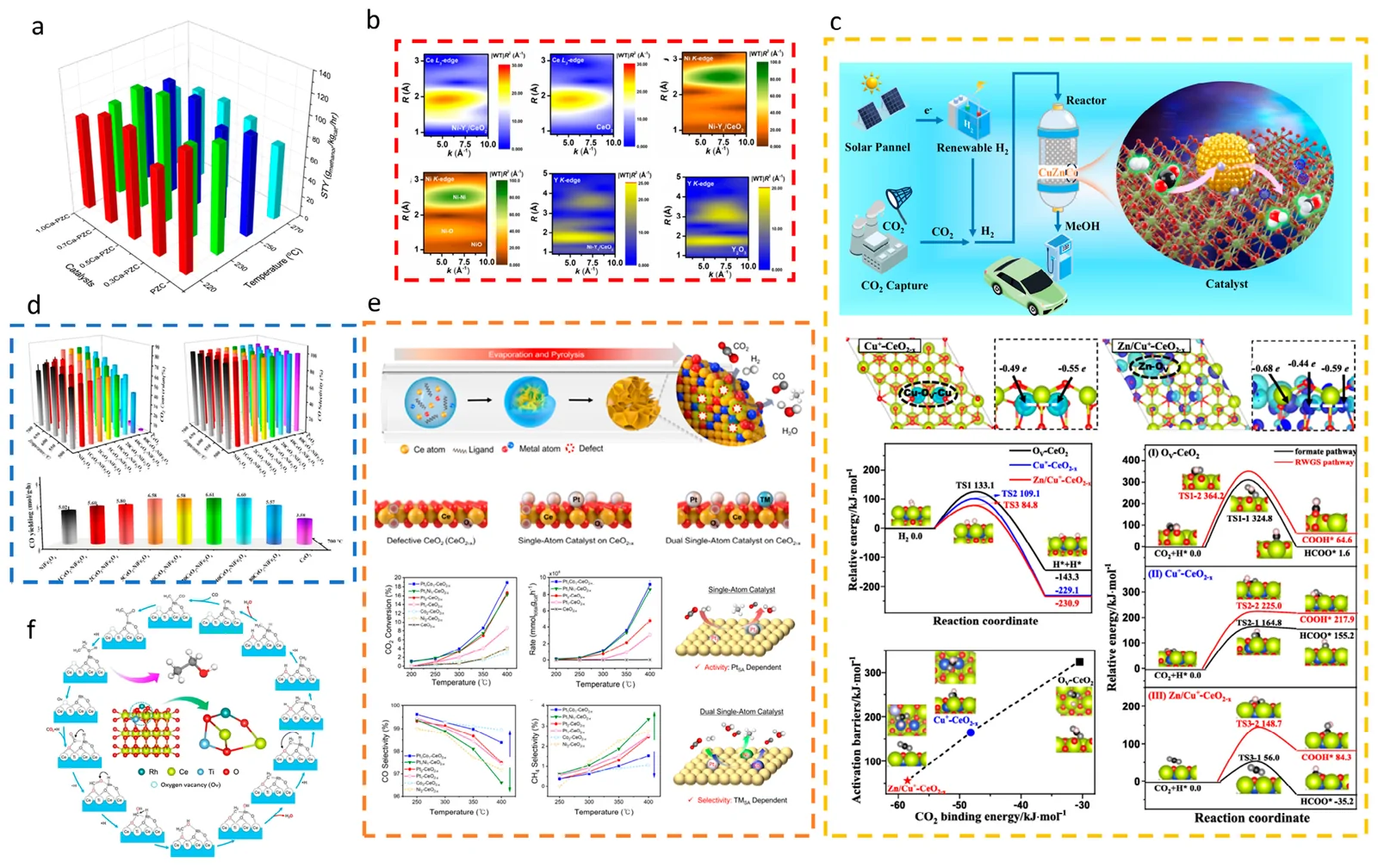
(**a**) Methanol STY (g Methanol/kg cat/hr) over % xCa-PdZn/CeO_2_ catalysts at different temperatures. Reprinted from Ref. [112], Copyright (2023), with permission from Elsevier. (**b**) Model catalysts for Ni-Y_x_/CeO_2_ at (A-B) Ce L_3_-edge, (C-E) Ni K-edge, (F-H) Y K-edge. Reprinted from Ref. [117], Copyright (2026), with permission from Elsevier (**c**) illustration of the thermal CO_2_ hydrogenation to methanol. DFT calculation results over the OV-CeO_2_,Cu+-CeO_2-x_ and Zn/Cu+-CeO_2-x_ catalysts to investigate the reaction mechanism. Reprinted from Ref. [116], under CC BY 4.0 license. (**d**) CO_2_ hydrogenation performance with CeO_2_-NiFe_2_O_4_ catalysts. CO_2_ conversion, CO product selectivity, and CO yielding of CeO_2_-NiFe_2_O_4_ at 700 °C. Reprinted from Ref. [118], Copyright (2024), with permission from Elsevier. (**e**) Schematic of particle synthesis. Concept of single-atoms and dual single-atoms on CeO_2 x_ support. Catalytic performance of Pt_1_-, Pt_2_-, Pt_1_Ni_1_-, and Pt_1_Co_1_-CeO_2_ x, CeO_2_ x, Ni_2_-CeO_2_ x, and Co_2_-CeO_2_ x during CO_2_ hydrogenation. Reprinted from Ref. [113], Copyright (2025), with permission from Elsevier. (**f**) The illustrated catalytic cycle of ethanol formation from CO_2_ hydrogenation on the Rh_1_/CeTiO_x_ catalyst. The inset gives the structure of Rh_1_/CeTiO_x_ catalyst. Reprinted from Ref. [95], Copyright (2022), with permission from Wiley-VCH.

PdZn/CeO_2_ is a typical system for bimetallic-promoted CO_2_-to-methanol. Malik et al. compared Pd, Zn, PdZn alloy, and Ca-doped PdZn/CeO_2_; Ca-doped PdZn/CeO_2_ achieved nearly 100% methanol selectivity and 7.7% CO_2_ conversion at 220 °C, 30 bar, GHSV = 2400 mL g^−1^ h^−1^; HR-TEM showed PdZn nanoparticles of 3–6 nm on CeO_2_, XPS confirmed Ce^3+^ and oxygen vacancies, and in situ DRIFTS detected formate, CH_2_O, and CH_3_O intermediates [100]. Subsequently, in Figure 6a, Zaman et al. further optimized Ca loading and found that 0.5 wt% Ca with PdZn alloy gave the best synergy, achieving 15.9–16% CO_2_ conversion, >93% methanol selectivity, and about 122–124 g kg_cat^−1^ h^−1^ methanol STY at 230 °C, 20 bar, GHSV = 2400 h^−1^; In the corresponding study, CO_2_ desorption measurements showed that the 0.5Ca-PZC catalyst exhibited a CO_2_ desorption amount of 66.90 μmol g_cat^−1^, higher than the 55.91 μmol g_cat^−1^ measured for undoped PZC, indicating that an appropriate Ca loading increases the density of basic sites and enhances CO_2_ adsorption [112]. The outstanding value of PdZn-Ca/CeO_2_ lies in showing that PdZn alloy provides the main active phase for methanol, while Ca is not simply a “basicity enhancer”; it enhances CO_2_ activation and H_2_ spillover through an appropriate Ce^3+^/Ce^4+^ ratio, basic sites, and oxygen vacancy concentration, thereby improving formate pathway efficiency. However, more Ca is not better; although 1.0Ca-PZC had the highest CO_2_ desorption (83.29 μmol g_cat^−1^), methanol selectivity dropped to 88.1% and STY to 90.7 g kg_cat^−1^ h^−1^, indicating that too strong or too many basic sites may promote side reactions or alter the PdZn-CeO_2_ interface balance [112].

The Pd-Cu/CeO_2_ system further reveals the importance of “moderate metal-support interaction.” Lin et al. compared Pd-Cu bimetallic catalysts supported on TiO_2_, ZrO_2_, CeO_2_, Al_2_O_3_, and SiO_2_, and found that Pd-Cu/TiO_2_-P1 and Pd-Cu/ZrO_2_ gave CO_2_ conversions of 16.4% and 15.8%, with methanol formation rates of 0.50 and 0.52 μmol g_cat^−1^ s^−1^, about 1.6 times that of Pd-Cu/SiO_2_; in contrast, Pd-Cu/CeO_2_-D, despite having the strongest metal-support interaction, performed poorly because the PdCu_3_ alloy reconstructed to PdCu_x_, reducing the proportion of weakly adsorbed CO_2_ and the surface H_2_/CO_2_ adsorption ratio [114]. This result is particularly cautionary for CeO_2_ systems: strong MSI does not necessarily favor methanol synthesis. When CeO_2_ interacts too strongly with Pd-Cu, it may destroy the alloy structure or weaken the simultaneous adsorption of CO_2_/H_2_, lowering methanol formation. Hence, in bimetallic/CeO_2_ design, “strong interaction” should not be the sole target; instead, a moderate coupling among PdCu or CuZn alloy/interface, oxygen vacancies, and H_2_ activation should be pursued.

CuNi/CeO_2_ demonstrates the impact of morphology control on bimetallic methanol synthesis. Tan et al. prepared Cu-Ni catalysts on CeO_2_ with different morphologies, finding that 100–300 nm CeO_2_ nanorods mainly expose (100)/(110) facets, while 10–20 nm nanospheres and irregular nanoparticles are mainly covered by (111) facets; 1Cu2Ni/CeO_2_-NR exhibited the highest Ce^3+^ concentration (26.7%, vs. 21.6% for NS and 21.4% for NP) and the highest O_vac ratio (46.7%, vs. 41.4% for NS and 40.8% for NP), thus outperforming nanospheres and nanoparticles in CO_2_-to-methanol [119]. This system shows that although Cu-Ni bimetallic provides H_2_ activation and CO_x_ intermediate hydrogenation, the exposed facets and oxygen vacancy number of CeO_2_ determine the initial CO_2_ activation.

W doping of Cu/CeO_2_ represents a route to regulate CeO_2_ redox activity and enhance methanol selectivity. Yan et al. pointed out that on unmodified Cu/CeO_2_, methanol typically forms via the RWGS-CO intermediate pathway, with apparent activation energies for CO and methanol formation both around 55 ± 5 kJ mol^−1^; on Cu/CeW_0_._25_O_x_, the apparent activation energies for methanol and CO formation were 38 ± 2 and 58 ± 2 kJ mol^−1^, respectively, indicating that after W doping, RWGS is no longer the rate-limiting step for methanol formation. At 250 °C, 35 bar, CO_2_/H_2_/N_2_ = 23/69/8, the methanol STY of Cu/CeW_0_._25_O_x_ increased from 1.26 mol MeOH kg_cat^−1^ h^−1^ for undoped Cu/CeO_2_ to 12.3 mol MeOH kg_cat^−1^ h^−1^, with CO_2_ conversion and methanol selectivity reaching 13% and 87%, respectively. CO_2_-TPD and NAP-XPS indicated that W doping stabilizes Ce^4+^ as Ce^3+^ while suppressing the overly strong redox activity and the MvK pathway of CO_2_ dissociation to CO, steering the reaction toward the formate/methoxy pathway and methanol formation [46].

The Ce-CuZn catalyst pushes the interface design of bimetallic/ceria to the atomic level. Ye et al., through atomic-scale substitution of Cu/Zn in a Ce-MOF precursor, constructed a Ce-CuZn catalyst rich in Cu/Zn-Oᵥ-Ce sites, achieving 71.1% methanol selectivity, a methanol STY of 400.3 g kg_cat^−1^ h^−1^ at 260 °C, and stable operation for 170 h; at 280 °C, 2.0 MPa, GHSV = 10,000 mL g_cat^−1^ h^−1^, the methanol STY of Ce-CuZn was 140.6 g kg_cat^−1^ h^−1^, with a TOF of 19.0 h^−1^, 5–8 times that of other CuZnCe series samples (2.3–3.4 h^−1^) [116]. In situ DRIFTS identified HCOO* as a dominant surface species. For the Zn/Cu^+^–CeO_2−x_ model, DFT calculations predict a lower barrier for HCOO* formation than for the corresponding Cu^+^–CeO_2−x_ and Oᵥ–CeO_2_ models. Together, these observations are consistent with a Zn-modified Cu^+^–Oᵥ–Ce ensemble favouring formate formation, but do not independently verify this ensemble as the sole active site [116].

#### 3.2.3. Methanation: H_2_ Dissociation-CO_x_ Hydrogenation Synergy in NiCo, NiFe, Ni-Y, and La-M-CeO_2_

Y single-atom regulation of Ni/CeO_2_ is another highly active methanation design. In Figure 6b, Choudhary et al. constructed Ni-Y_1_/CeO_2_, where Ni exists as nanoparticles and Y^3+^ is atomically dispersed on CeO_2_; this catalyst achieved 83% CO_2_ conversion and 100% CH_4_ selectivity at 350 °C, and also exhibited superior low-temperature activity over Ni/CeO_2_ and impregnated NiY/CeO_2_-imp in the 250–300 °C range, with good 40-h stability. Y^3+^ single atoms increased the surface area of Ni-Y_1_/CeO_2_ from 22 m^2^ g^−1^ for Ni/CeO_2_ to 34 m^2^ g^−1^, and raised total basic sites to 34.14 μmol g^−1^, significantly higher than 18.36 μmol g^−1^ for Ni/CeO_2_ and 23.28 μmol g^−1^ for NiY/CeO_2_-imp [117]. Similarly, Eu^3+^ doping of Ni/CeO_2_ can inhibit grain growth of CeO_2_ and NiO, improve Ni dispersion, and generate more Ni-O-Ce interfaces, lowering the onset temperature for complete CO_2_ conversion from 230 °C for Ni/CeO_2_ to 210 °C for Ni/CeEu(9:1) [120]. These results collectively indicate that the main role of rare-earth single atoms or heterovalent doping is to reshape Ni-CeO_2_ contact, basic sites, and oxygen vacancies, rather than replacing Ni’s deep- hydrogenation function.

The Ce-CuZn catalyst further advances bimetallic synergy to atomic-level Cu/Zn-Oᵥ-Ce active site design. In Figure 6c, differential charge density and potential energy profiles show that Zn/Cu^+^-CeO_2−x_ sites can significantly promote CO_2_ hydrogenation to HCOO*: the barriers for CO_2_ hydrogenation to HCOO* on Oᵥ-CeO_2_, Cu^+^-CeO_2−x_, and Zn/Cu^+^-CeO_2−x_ are 324.8, 164.8, and 56.0 kJ mol^−1^, respectively, and the HCOO* pathway is thermodynamically and kinetically superior to the COOH* pathway [116]. This indicates that Zn addition does not simply dilute Cu sites, but together with Cu^+^ and CeO_2_ oxygen vacancies enhances CO_2_ binding, lowers the formate formation barrier, and suppresses the RWGS branch via COOH*. Thus, this system achieves 71.1% methanol selectivity and a methanol STY of 400.3 g kg_cat^−1^ h^−1^ at 260 °C with 170-h stability, demonstrating the directed strengthening of the Cu-CeO_2_ methanol pathway through bimetallic doping.

La-M-CeO_2_ (M = Sm, Pr, Mg) as a composite doping support for Ni emphasizes the influence of rare-earth/alkaline-earth co-doping on sintering resistance, oxygen vacancies, and methanation stability. Siakavelas et al., using H_2_-TPR, H_2_-TPD, Raman, XPS, and TEM, pointed out that La-M composite oxides increase oxygen vacancies, tune Ni reducibility, and improve sintering resistance, leading to highly selective and stable CO_2_ methanation performance [121]. Studies on alkaline-earth-modified Ni/M_0_._1_Ce_0_._9_O_x_ (M = Mg, Ca, Sr, Ba) further showed that MgO and CaO tend to enter the CeO_2_ lattice to form solid solutions, while SrO and BaO are more dispersed as carbonates on the surface; the catalytic activity order was Ni/Ca_0_._1_Ce_0_._9_O_x_ > Ni/Sr_0_._1_Ce_0_._9_O_x_ > Ni/Mg_0_._1_Ce_0_._9_O_x_ > Ni/Ba_0_._1_Ce_0_._9_O_x_ > Ni/CeO_2_ [122]. This suggests that moderate basic sites and surface oxygen vacancies favor CO_2_ activation, but too strong or poorly positioned basic sites do not necessarily enhance methanation. Similarly, in situ XAS and NAP-XPS on Ni/La-doped CeO_2_ showed that La incorporation into the CeO_2_ lattice increases oxygen mobility and causes surface/bulk Ce^3+^ to continuously reconstruct during reaction [123].

Low-loaded Ni-M and Ru-M (M = Co, Mn) systems indicate that the second metal and support thermal properties are also important for high-temperature methanation. Wasnik et al. compared CeO_2_- and SiC-supported 2 wt% Ni/Ru with 2 wt% Co/Mn modified bimetallic catalysts; at 500 °C, atmospheric pressure, H_2_/CO_2_ = 4, total flow 70 mL min^−1^, GHSV = 12,000 h^−1^, 20 mg of 2Ni-2Co/SiC achieved 77% CO_2_ conversion and 88% CH_4_ selectivity; under the same conditions, 2Ni-2Co/CeO_2_ achieved 76% CO_2_ conversion and 83% CH_4_ selectivity, with only 3–5% decay over 40 h [115]. This work shows that CeO_2_’s CO_2_ affinity and oxygen vacancies favor low-loaded Ni-Co methanation, but in high-temperature exothermic reactions, SiC’s high thermal conductivity can also improve stability by mitigating hot spots and heat transfer limitations.

#### 3.2.4. RWGS and CO Selectivity: NiFe Alloy Confinement, La Doping, and Selective CO Methanation

CeO_2_-confined NiFe is a typical example of stabilizing bimetallic alloys for high-temperature RWGS. In Figure 6d, Lu et al. used CeO_2_-NiFe_2_O_4_ composite catalysts to study high-temperature CO_2_ hydrogenation to CO at 700 °C; pure NiFe_2_O_4_ deactivated rapidly after reduction to Fe-Ni alloy, whereas CeO_2_ addition significantly improved activity and stability; 20CeO_2_-NiFe_2_O_4_ achieved 80.85% CO_2_ conversion at 700 °C and remained stable for 50 h. CeO_2_ confinement maintained smaller Ni-Fe alloy crystallites and higher surface area, providing more active sites; simultaneously, CeO_2_ introduced higher oxygen vacancy concentrations, promoting CO_2_ activation and formate formation, and suppressing excessive Fe-Ni alloy aggregation and coking through CeO_2_/CeFeO_3_ dispersion [118].

This NiFe-CeO_2_ result contrasts sharply with methanation systems: again a Ni-based bimetallic, if the Fe-Ni alloy is coupled with CeO_2_ redox cycling at high temperature, the product outlet can be biased toward CO, whereas Ni-Y/CeO_2_ at lower temperatures and stronger H_2_ dissociation/deep hydrogenation favors CH_4_ [115,117,118]. Therefore, the product selectivity of bimetallic CeO_2_ should be discussed jointly in terms of “metal hydrogenation ability + CeO_2_ oxygen vacancy activity + reaction temperature,” rather than judged solely by metal type.

Although not a typical bimetallic system, the selective CO methanation over Ni(Cl)/CeO_2_ provides important supporting evidence for “second-regulating factor selectively blocking CO_2_ hydrogenation on CeO_2_.” Konishcheva et al. found that on Ni(Cl)/CeO_2_, the CO methanation rate increases with H_2_ concentration, is independent of CO_2_ concentration, and decreases with increasing CO/H_2_O; CO_2_ methanation is weakly sensitive to H_2_/CO_2_ concentration, and CO_2_ conversion does not exceed 6% in the 180–320 °C range. The authors attributed the high selectivity to strongly adsorbed CO blocking the Ni surface and Cl blocking the CeO_2_ surface, jointly inhibiting CO_2_ hydrogenation. Structured Ni(Cl)/CeO_2_-η-Al_2_O_3_/FeCrAl wire-mesh catalysts achieved deep CO removal from formic-acid-derived H_2_-rich gas at 180–220 °C and WHSV up to about 200 L(STP) g^−1^ h^−1^, with selectivity of 30–70% [124].

#### 3.2.5. Dual Single Atoms and Single-Atom-Modified CeO_2_: Shrinking Metal Synergy to the Atomic Scale

Dual-single-atom catalysts provide a clearer atomic-scale model for bimetallic/CeO_2_ synergy. In Figure 6e, Park et al., using aerosol-assisted spray pyrolysis, prepared Pt, Co, Ni single atoms and Pt/Co, Pt/Ni dual single atoms on mesoporous defective CeO_2−x_ for CO_2_ hydrogenation. EXAFS showed a Pt-O peak at about 1.7 Å for all SAC/DSAC samples, confirming Pt atomic dispersion; Pt_1_Co_1_-CeO_2−x_ and Pt_1_Ni_1_-CeO_2−x_ also showed Pt-Co or Pt-Ni interaction peaks at about 2.2 Å, indicating electronic metal-metal interactions between the dual atoms [113]. In CO_2_ hydrogenation, Co_2_-CeO_2−x_ and Pt_1_Co_1_-CeO_2−x_ exhibited CO selectivity above 98% at all tested temperatures, while Ni-containing catalysts tended to increase CH_4_ selectivity; this indicates that Pt mainly affects the number of active sites and H_2_/CO_2_ activation, while Co/Ni as the second single atom regulates the CO/CH_4_ pathway splitting [113].

Ti-doped CeO_2_ stabilizing Rh single atoms demonstrates the extension of dual-component regulation toward C_2_ oxygenates. In Figure 6f, the mechanistic scheme for Rh_1_/CeTiO_x_ emphasizes the Ti-doping-induced oxygen vacancy-Rh Lewis acid-base pair: Based on the in situ spectroscopic results and DFT analysis, an Oᵥ–Rh Lewis acid-base pair has been proposed for Rh_1_/CeTiO_x_. In this model, oxygen vacancies interact with CO_2_-derived or oxygenated intermediates, whereas adjacent Rh single atoms stabilize carbon-containing intermediates and may facilitate steps leading to C–C coupling [95]. DFT results show that the barrier for CO* + CH_x_* coupling to CH_x_CO* is only 0.48 eV, lower than the 1.48 eV for CO* hydrogenation to CHO*; simultaneously, Rh single atoms, compared to Rh clusters, are more favorable for stabilizing CO* and avoiding its over-hydrogenation to methanol or methane. Thus, Rh_1_/CeTiO_x_ achieves about 99.1% ethanol selectivity and a TOF of 493.1 h^−1^, indicating that stabilizing single atoms via support doping and constructing Oᵥ-Rh acid-base pairs is an important strategy for shifting CeO_2_-based catalysts from C_1_ products to C-C coupling products.

Ti-doped CeO_2_ stabilizing Rh single atoms also provides an extreme selectivity case for “doped CeO_2_ + noble-metal single atom” in CO_2_ hydrogenation to ethanol. Zheng et al. reported that the Rh_1_/CeTiO_x_ single-atom catalyst exhibits about 99.1% ethanol selectivity, a TOF of 493.1 h^−1^, and excellent stability. Ti doping promotes oxygen vacancy formation, constructing oxygen vacancy-Rh Lewis acid-base pairs that favor CO_2_ adsorption/activation, C-O bond cleavage in CH_x_OH* and COOH*, and subsequent C-C coupling and hydrogenation to ethanol; meanwhile, Ti doping induces lattice reconstruction and forms strong Rh-O bonds to stabilize Rh single atoms [95]. Fe and Na co-modification of Rh/CeO_2_ can also be classified under this logic: Fe/Na promotes Rh dispersion and Rh^+^ species formation, enhancing CO_2_ adsorption/dissociation, so that 2Rh0.5Fe0.5Na/CeO_2_ achieves 13.0% CO_2_ conversion, 29.8% ethanol selectivity, and an ethanol production rate of 116.7 mmol g_Rh^−1^ h^−1^ at 250 °C, 3 MPa [125].

These two atomic-scale systems can be seen as advanced directions for bimetallic doping: conventional bimetallic alloys emphasize electronic/geometric effects in metal particles, while dual-single-atom and doped-stabilized single-atom systems emphasize the local coordination structure among isolated metal sites, CeO_2_ oxygen vacancies, and the second atom. The common lesson from Pt-Co, Pt-Ni, and Rh_1_/CeTiO_x_ is that the second metal or heterovalent dopant does not simply increase activity, but selects CO, CH_4_, or ethanol pathways by modifying local coordination, electron transfer, and oxygen vacancy proximity. The challenge is whether single atoms agglomerate, whether the second metal migrates under reaction conditions, and how the true dynamic active sites can be confirmed in situ [95,113].

Table 3 is organized according to the target product to facilitate comparison within related reaction classes. CO-selective systems, including Ni–In, Pt–Co dual-single-atom, and La-modified Ni/CeO_2_ catalysts, generally maintain high CO selectivity under their respective reported conditions. Ni–Y, Ni–Co, Ni–Mn, and La/Sm-modified Ni systems are more commonly associated with CH_4_ formation, whereas Cu–Ni, PdZn, Cu–Zn, and related systems are mainly investigated for methanol synthesis. Rh–Fe–Na/CeO_2_ further extends the product range toward C_2_ oxygenates. These results suggest that the second metal can regulate electron transfer, H_2_ activation, local coordination, surface basicity, and oxygen-vacancy chemistry. However, the magnitude of the reported performance should not be compared across reaction classes without considering the differences in feed composition, pressure, space velocity, reactor configuration, and performance normalization.

**Table 3:** Reported catalytic performance of multimetal-modified CeO_2_ catalysts, organized by target reaction.

Catalyst	Product	Temperature (°C)	Pressure (MPa)	GHSV/Space Velocity	Conversion (%)	Selectivity (%)	TOF/STY (Type and Units)	Stability Test Duration (h)	Ref.
NiIn(4)/CeO_2_	CO	370 °C	0.1	5000 mL·gcat^−1^·h^−1^	23.9 (CO_2_)	99.1 (CO)	STY: 26.1 mmol/g/h	>100 h	[89]
Pt_1_Co_1_-CeO_2−x_	CO	350 °C	0.6	-	~60 (CO_2_)	~99 (CO)	-	11 h	[113]
NiCe_0_._9_La_0_._1_	CO	600 °C	0.1	300,000 mL·gcat^−1^·h^−1^	-	~95 (CO)	-	22 h	[123]
NiMn(4)/CeO_2_	CH_4_	370 °C	0.1	5000 mL·gcat^−1^·h^−1^	35.3 (CO_2_)	86.6 (CH_4_)	STY: 33.8 mmol/g/h	>100 h	[89]
2Ni-2Co/CeO_2_	CH_4_	500 °C	0.1	12,000 mL·gcat^−1^·h^−1^	~76 (CO_2_)	83 (CH_4_)	-	40 h	[115]
Ni-Y_1_/CeO_2_	CH_4_	350 °C	0.1	30,000 mL·gcat^−1^·h^−1^	83 (CO_2_)	~100 (CH_4_)	STY: 265 mmol gcat^−1^ h^−1^	100 h	[117]
Ni/La-Sm-Ce	CH_4_	350 °C	0.1	25,000 mL·gcat^−1^·h^−1^	~53 (CO_2_)	~100 (CH_4_)	TOF: (300 °C): 54 h^−1^	20 h	[121]
1Cu2Ni/CeO_2_-NR	CH_3_OH	260 °C	3.0	6000 mL·gcat^−1^·h^−1^	18.35 (CO_2_)	73.33 (CH_3_OH)	-	100 h	[119]
Ce-CuZn	CH_3_OH	260 °C	2.0	10,000 mL·gcat^−1^·h^−1^	~8.0 (CO_2_)	71.1 (CH_3_OH)	STY: 12.5 mmol_MeOH·g_cat^−1^·h^−1^	170 h	[116]
0.5Ca5P5ZC (PdZn/CeO_2_)	CH_3_OH	220 °C	3.0	2400 mL·gcat^−1^·h^−1^	7.7 (CO_2_)	100 (CH_3_OH)	-	62 h	[100]
PZC (PdZn/CeO_2_)	CH_3_OH	220 °C	2.0	2400 mL·gcat^−1^·h^−1^	14.1 (CO_2_)	~95 (CH_3_OH)	STY: 5.15 mmol_MeOH·g_cat^−1^·h^−1^	-	[112]
Cu/Ce_0_._9_Zn_0_._1_O_2_	CH_3_OH	280 °C	3.0	24,000 mL·gcat^−1^·h^−1^	12.1 (CO_2_)	93.8 (CH_3_OH)	TOF: > 360 h^−1^	-	[126]
Cu/CeW_0_._25_O_x_	CH_3_OH	250 °C	3.5	15,000 mL·gcat^−1^·h^−1^	13.0 (CO_2_)	87 (CH_3_OH)	STY: 12.32 mmol_MeOH·g_cat^−1^·h^−1^	72 h	[46]
CuCeZr	CH_3_OH	240 °C	3.0	20,000 mL·gcat^−1^·h^−1^	~8 (CO_2_)	~70 (CH_3_OH)	-	100 h	[46]
2Rh0.5Fe0.5Na/CeO_2_	C_2_H_5_OH	250 °C	3.0	4000 mL·gcat^−1^·h^−1^	13.0 (CO_2_)	29.8 (C_2_H_5_OH)	STY: 116.7 mmol·(gRh)^−1^·h^−1^	50 h	[125]

**Note:** Unit conversions were applied only when the conversion was unambiguous. TOF values are expressed in h^−1^, while STY values are expressed in mmol of product per gram of the reported catalyst or metal basis per hour where conversion was possible. The original normalization basis was retained. A dash (-) indicates that the corresponding value was not reported. The values are not directly comparable across different feed compositions, pressures, space velocities, and catalyst bases.

Thus, the key to bimetallic design is to clarify the role of the second component: it can stabilize Ce^3+^, modulate oxygen vacancy activity, change metal electronic structure, or construct single-atom coordination environments, but these effects must match the rate-determining step of the target product. Otherwise, increasing defects or enhancing metal-support interaction does not necessarily lead to higher target selectivity. Therefore, the evaluation criteria for bimetallic/CeO_2_ should shift from “whether conversion is increased” to “whether the pathway branching point is altered.” If the second metal only improves metal dispersion or total CO_2_ adsorption, its role remains at the promoter level; only when it can change the relative stability of key intermediates such as COOH*, formate, CO*, CH_x_*, or methoxy does it constitute truly explainable selectivity control. Bimetallic and composite oxide systems offer viable pathways to balance activity, selectivity, and stability for industrial deployment. For methanol synthesis, PdZn and CuZn systems improve selectivity beyond single-metal Cu/CeO_2_, reducing downstream purification costs, though noble metal loadings must be minimized for economic viability. For high-temperature RWGS, NiFe–CeO_2_ composites combine high CO selectivity with thermal stability, suitable for integration with Fischer–Tropsch units for liquid fuel production [71]. Integrated capture-conversion systems based on MgO–CeO_2_ eliminate the need for separate CO_2_ purification units, enabling direct utilization of dilute flue gas streams [127]. The primary barrier to industrial translation remains the scalability of controlled interfacial structures and their long-term durability under realistic process conditions [14].

### 3.3. Ceria–Metal Oxide Mixed Oxides

The interfaces formed between CeO_2_ and other oxides further expand the design space for CO/CO_2_ hydrogenation. MgO and CaO mainly provide CO_2_ capture, basic sites, and carbonate reservoirs, suitable for integrated capture-conversion systems; ZrO_2_ is more commonly used to tune oxygen vacancy stability, water tolerance, and Cu/In-based methanol pathways; ZnO-CeO_2_ combinations promote methanol selectivity in Cu-based systems while potentially inhibiting deep hydrogenation in Ni-based systems. NiO, CoO_x_, and FeO represent variable-valence oxide interfaces whose reactive structures may reconstruct under H_2_/CO_2_ switching. In_2_O_3_/CeO_2_ demonstrates that pure oxide interfaces can also generate methanol via the formate pathway, but water-induced hydroxylation and support sintering remain important limitations. Thus, the goal of composite oxide design is not to maximize any single characterization parameter, but to maintain a sustainable balance among CO_2_ capture, oxygen migration, intermediate conversion, and water removal [104,128,129,130,131,132,133,134,135]. For such materials, it is particularly necessary to report both adsorption and catalysis metrics simultaneously. High CO_2_ capture capacity does not guarantee that the captured carbonate can be rapidly converted in hydrogen; strong CO_2_ adsorption may also cause site blocking. Conversely, weaker but reversible adsorption sites, regenerable redox vacancies, and fast water desorption are sometimes more favorable for stable turnover. Therefore, the performance evaluation of composite oxide/CeO_2_ should include cycling capacity, post-reaction phase structure, deactivation pathways, and capture-conversion coupling efficiency.

#### 3.3.1. Core Logic of Composite Oxide/CeO_2_: Separating CO_2_ Adsorption, Redox Cycling, and Hydrogenation Sites

The essence of synergy between CeO_2_ and other metal oxides is not simply replacing CeO_2_ with another support, but splitting the key functions in CO_2_ hydrogenation through composite oxide interfaces: MgO/CaO provide CO_2_ capture and basic sites; ZrO_2_ and ZnO tune the methanol pathway of Cu or In_2_O_3_; NiO/CoO_x_/FeO form variable-valence oxide interfaces with CeO_2_ for CH_4_ or CO production; and binary supports of TiO_2_, ZrO_2_, and CeO_2_ modulate Pd-Cu or Cu-Ga methanol synthesis through moderate SMSI and weak CO_2_ adsorption species [128,134,135,136,137,138,139].

Compared with single-metal/CeO_2_, the most noteworthy aspect after introducing other metal oxides is “interfacial functional partitioning”: CeO_2_ is responsible for Ce^3+^/Ce^4+^ cycling, oxygen vacancies, and H spillover; the second oxide is responsible for basicity, hydrophobicity/hydrophilicity, lattice oxygen migration, metal dispersion, or CO_2_ capture. When this partitioning is rational, the reaction can be steered toward highly selective methanol, methane, or CO; when unbalanced, the hydrophilicity, overly strong redox properties, or excessive metal encapsulation of CeO_2_ can lead to deactivation or side reactions [111,129,136,140,141].

#### 3.3.2. MgO/CaO-CeO_2_: Adsorption-Catalysis Coupling in Integrated CO_2_ Capture and In Situ Methanation/Conversion

**Figure 7:**
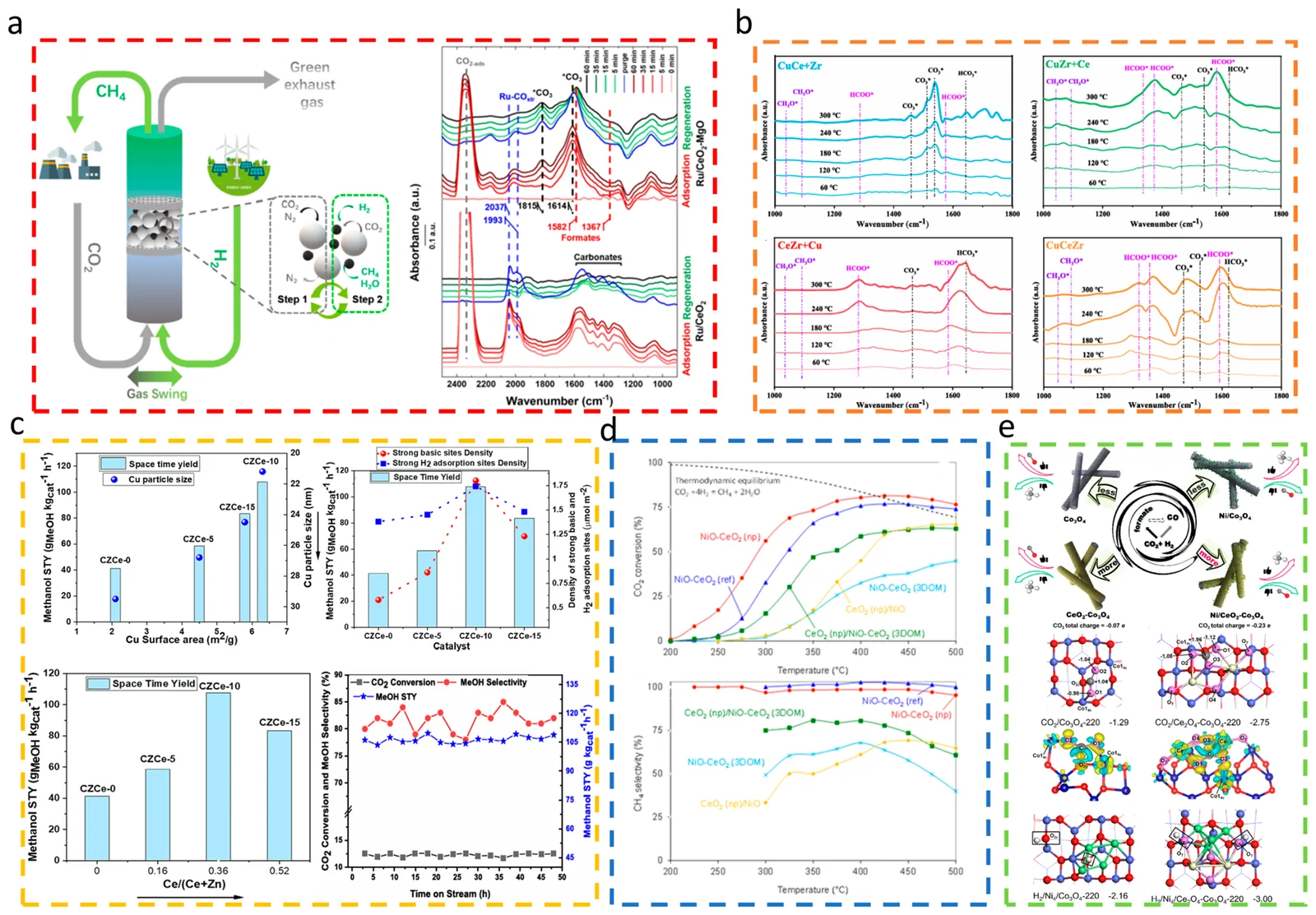
(**a**) Schematic diagram of integrated CO2 capture and methanation process. In situ DRIFTS of ICCM over Ru/rod-CeO_2_-MgO and Ru/rod-CeO_2_. Reprinted from Ref. [128], under CC BY 4.0 license. (**b**) In situ DRIFT spectra of CO_2_ + H_2_ on CuCe + Zr, CuZr + Ce, CeZr + Cu, and CuCeZr. Reprinted from Ref. [140], Copyright (2025), with permission from Elsevier. (**c**) Structure activity correlations methanol STY and Cu SA and Cu particle size MeOH STY and strong basic site density and strong H methanol STY and Ce/(Ce + Zn) ratio. Time on Stream for CZCe-10 catalyst at 250 °C, 30 bar, 3000 mL·g^−1^·h^−1^ and CO_2_:H_2_ ratio 1:3. Reprinted from Ref. [133], Copyright (2024), with permission from Elsevier. (**d**) CO_2_ methanation catalytic experiments (200 mg of catalyst; 200 mL/min; CO_2_, H_2_, and N_2_ in 16:64:20%v proportion; Pre-treatment in 50% H_2_/N_2_ at 500 °C). Reprinted from Ref. [130], Copyright (2021), with permission from Elsevier. (**e**) Optimized structures of adsorbed CO_2_ on perfect and Ce_2_O_4_ supported Co_3_O_4_-220 surfaces with corresponding charge density difference plots, and adsorbed H_2_ on Ni_4_ supported and both Ni_4_ and Ce_2_O_4_ supported Co_3_O_4_-220 surfaces. Reprinted from Ref. [139], Copyright (2023), with permission from Elsevier.

The combination of MgO and CeO_2_ is often used for integrated CO_2_ capture and methanation (ICCM). Sun et al. constructed Ru/CeO_2_-MgO composite materials, where Ru/CeO_2_ handles methanation and Li/Na/K-doped MgO handles CO_2_ capture, systematically comparing CeO_2_ nanorods, particles, and cubes. Compared with cube-CeO_2_, rod-CeO_2_ and particle-CeO_2_ had better Ru dispersion and stronger SMSI; Ru/rod-CeO_2_-MgO and Ru/particle-CeO_2_-MgO gave CH_4_ yields of 0.33 and 0.29 mmol g^−1^, and CO_2_ conversions of 55.7% and 59.8%, respectively, significantly outperforming Ru/cube-CeO_2_-MgO, and Ru/rod-CeO_2_-MgO maintained good stability over 9 ICCM cycles. In Figure 7a, in situ DRIFTS indicated that formate and Ru-CO are key methanation intermediates in ICCM [128].

CaO-CeO_2_ systems are more oriented toward high-temperature CO_2_ capture or capture-conversion coupling. Fan et al. studied CaO-Cu/CeO_2_ for integrated CO_2_ capture and in situ conversion to CO, pointing out that CeO_2_ can both suppress CaO sintering and influence RWGS performance through oxygen vacancies; CeO_2_ morphology changes the number of oxygen vacancies, CaO dispersion, and Cu-CeO_2_ contact, thereby affecting CO production in ICCC-RWGS [142]. Wu et al. designed (CaO/CeO_2_)@CeO_2_ composite adsorbents via a microinjection titration-calcination strategy to improve CO_2_ adsorption stability; Li et al. further doped CaO/CeO_2_ with La, Gd, and Pr, and DFT showed that rare-earth doping lowers oxygen vacancy formation energy, promoting CO_2_ diffusion and O^2−^ migration, thus improving high-temperature cyclic capture performance [143,144]. Although these works are not directly CO_2_ hydrogenation reactions, they serve as supporting evidence for “CeO_2_-alkaline-earth oxide composite interfaces enhancing CO_2_ capture, sintering resistance, and oxygen migration.”.

#### 3.3.3. ZrO_2_/CeO_2_: Stabilizing Oxygen Vacancies, Moderate MSI, and Water-Resistance in Methanol Synthesis

The combination of ZrO_2_ and CeO_2_ is one of the most common oxide synergy strategies in CO_2_-to-methanol. Sharma et al. compared In_2_O_3_/ZrO_2_ and In_2_O_3_/CeO_2_ and found that although In loadings and surface areas were similar, ZrO_2_ support improved In_2_O_3_ methanol synthesis activity and remained stable for 50 h, while In_13_/CeO_2_ deactivated after 2 h. The deactivation was attributed to the hydrophilicity and redox properties of CeO_2_ and its sintering in the presence of water; XPS and DRIFT showed significant formation of In(OH)_3_ and OH species on spent In_13_/CeO_2_, and Ar flushing only partially regenerated it [136]. This provides an important counterexample: the strong redox character and hydrophilicity of CeO_2_ are not always beneficial for methanol synthesis; water-induced hydroxyl accumulation and support sintering may make In_2_O_3_/CeO_2_ more prone to deactivation than In_2_O_3_/ZrO_2_.

Cu-CeO_2_-ZrO_2_ demonstrates the positive effect of strong MSI between Cu and CeO_2_-ZrO_2_ mixed oxides on methanol synthesis. In Figure 7b, Han et al. prepared CuCe+Zr, CuZr+Ce, CeZr+Cu, and one-step solid-state CuCeZr, finding that CuCeZr exhibited the best CO_2_ hydrogenation activity and stability, even surpassing commercial Cu-ZnO-Al_2_O_3_ syngas-to-methanol catalysts. Its high activity was attributed to the largest metallic Cu surface area and the most abundant Cu-support interfacial Cu species, both originating from the strongest MSI between Cu and CeO_2_-ZrO_2_; in situ DRIFTS showed that strong MSI promotes formate formation and rapid conversion to methoxy, enhancing methanol production efficiency [140]. Poto et al. performed kinetic modeling of CO_2_/CO hydrogenation to methanol over CuO/CeO_2_/ZrO_2_, emphasizing the combined role of Ce-Zr oxide oxygen vacancies and CO/CO_2_ hydrogenation pathways, providing rate-equation and pathway-level support for the Cu-Ce-Zr system [145].

In commercial Cu-ZnO-based methanol catalysts, ZrO_2_ and CeO_2_ also have different promotional effects. Esmaeili et al., under unified coprecipitation conditions, compared Al_2_O_3_, ZrO_2_, and CeO_2_ promoters for Cu-ZnO, finding that ZrO_2_ was more effective than Al_2_O_3_ or CeO_2_ in improving CO_2_ conversion and methanol yield; CZZC (Cu-ZnO-ZrO_2_-CeO_2_) at 250 °C, H_2_/CO_2_ = 3/1, GHSV = 5700 NmL g^−1^ h^−1^, and 4.5 MPa achieved 28.7% CO_2_ conversion, 44.3% methanol selectivity, and 12.74% methanol yield, representing improvements of about 18%, 33%, and 57% over CZA, respectively [134]. Damma et al. studied Cu-Ga supported on CeO_2_-ZrO_2_ and found that 1Cu1Ga/CZ(80:20) achieved a maximum CO_2_ conversion of 15% at 300 °C, 410 psi, and remained stable for 100 h at 240 °C, 410 psi, 4500 mL h^−1^ g^−1^, with CO_2_ conversion of 7.2% and methanol selectivity of 50% [145].

#### 3.3.4. ZnO/CeO_2_: Dual Role in Cu-Based Methanol Synthesis and Ni-Based CO/CH_4_ Splitting

The synergy of ZnO-CeO_2_ with Cu is particularly prominent in methanol synthesis. Zhu et al., using flame spray pyrolysis to prepare Cu/ZnO-CeO_2_, found that CeO_2_ enhances Cu dispersion compared with ZnO, and the ternary Cu/ZnO-CeO_2_ exhibited significantly higher methanol selectivity than binary Cu/CeO_2_ and Cu/ZnO. Cu-ZnO interaction forms Zn-modified Cu active sites promoting CO_2_-to-methanol, while Cu-CeO_2_ interaction suppresses RWGS through high formate coverage and faster CO intermediate hydrogenation. For low Cu loading samples, a small amount of ZnO (y ≤ 0.05) more than doubled the CH_3_OH formation rate; the CO formation rate over Ce-containing catalysts was lower than over Cu/ZnO, indicating that CeO_2_ suppresses RWGS [134].

Singh et al. systematically optimized the Ce/Zn ratio in Cu-ZnO-CeO_2_ and established relationships among strong basic site density, H_2_ adsorption sites, and methanol activity; in Figure 7c, at 250 °C, 30 bar, 3000 mL g_cat^−1^ h^−1^, the optimized catalyst achieved a methanol STY exceeding 106.7 g_MeOH kg_cat^−1^ h^−1^ [133]. Orona-Návar et al. compared CuCe, CuMg, CuCeMg, and CuZnAl, finding that CuCeMg achieved a methanol STY of 138 g_CH_3_OH kg_cat^−1^ h^−1^ at 230 °C, 3 MPa; CuCeMg combines the CO_2_ activation/redox capability of CeO_2_ with the medium-strong basic sites of MgO, exhibiting good low-temperature methanol selectivity and CO_2_ activation [131]. The morphology of CeO_2_ itself also alters the copper species at the CuO_x_-CeO_2_ interface. Wang et al. compared CuO supported on nanorods, nanocubes, and hydrangea-like CeO_2_; CuO/hydrangea-like-CeO_2_ achieved 15.6% CO_2_ conversion and 92.4% methanol selectivity at 260 °C, 3 MPa; its advantage came from a higher proportion of small-sized copper species favoring H_2_ activation, while Cu^2+^-Oᵥ-Ce_x_ species promoted oxygen vacancy and hydroxyl generation, thus favoring formate formation and hydrogenation to methanol. In situ DRIFTS showed that monodentate formate and bidentate formate are both key intermediates, with the main pathway being m-HCOO* → b-OCH_3_* → CH_3_OH [60].

The effect of ZnO on Ni/CeO_2_ is opposite, as it can significantly change CO/CH_4_ selectivity. Varvoutis et al. studied the effect of ZnO doping of CeO_2_ nanorods on Ni/CeO_2_ and found that Ni/CeO_2_ was almost completely selective to CH_4_, while Ni/ZnO and Ni/ZnO-CeO_2_ were clearly more favorable for CO formation at T < 450 °C; they attributed this to the suppression of key descriptors such as redox properties, basicity, and CO adsorption affinity by ZnO [111]. The same team also used Cs doping to tune the RWGS performance of CuO/CeO_2_, exploring the effect of 0–4 atoms Cs nm^−2^ on surface chemistry, CO_2_-TPD, XPS, FTIR, and CO_2_ hydrogenation selectivity, showing that alkali metals can finely tune CO selectivity by altering acid-base properties and Cu-CeO_2_ interfacial electronic structure [141].

Therefore, the role of ZnO-CeO_2_ must be distinguished by the main metal: in Cu-based systems, ZnO often promotes methanol through Zn-decorated Cu; in Ni-based systems, ZnO may weaken deep-hydrogenation ability, shifting the product from CH_4_ to CO [111,134,141].

#### 3.3.5. NiO, CoO_x_, FeO with CeO_2_: Variable-Valence Oxide Interfaces from Methanation to CO Selectivity

NiO-CeO_2_ mixed oxides are typical reducible oxide interfaces for CO_2_ methanation. In Figure 7d, Cárdenas-Arenas et al. prepared 6–7 nm NiO-CeO_2_ mixed oxide nanoparticles and found that their CO_2_ methanation rate at 275 °C was about 3 times that of an uncontrolled-particle-size reference sample, with near-100% CH_4_ selectivity up to 500 °C and maintained activity/selectivity over 25 h; the high activity was attributed to a high surface area of 122 m^2^ g^−1^ and highly reducible Ni-O-Ce species on the surface [130]. LaNiO_3_/CeO_2_-derived catalysts further demonstrate the advantage of perovskite-CeO_2_ interfaces. Onrubia-Calvo et al. reported that a 10% LaNiO_3_/CeO_2_-derived catalyst gave a TOF of 75.5 h^−1^ and CH_4_ selectivity > 92%, reaching 50% CO_2_ conversion at 315 °C and stable for 72 h at 325 °C; NAP-XPS showed that during reduction, the Ce^3+^ fraction increased from 16% to 41%, and Ni^0^ became more enriched at the surface, indicating that NiO-CeO_2−x_ interfaces and H_2_ spillover promote CeO_2_ surface reduction [146].

The three-component synergy in Ni/CeO_2_-Co_3_O_4_ further illustrates that composite oxides are not merely inert supports, but can distribute CO_2_ adsorption, H_2_ activation, and product selectivity among different components. In Figure 7e, the authors proposed that Co_3_O_4_ can adsorb and convert part of CO_2_, and CeO_2_-Co_3_O_4_ has stronger CO_2_ adsorption due to oxygen vacancies, but pure Co_3_O_4_ or CeO_2_-Co_3_O_4_ still mainly favors CO; after introducing Ni, the CH_4_ signal over Ni/CeO_2_-Co_3_O_4_ significantly increases, indicating that Ni plays a key role in H_2_ activation and deep hydrogenation to CH_4_ [139]. DFT results support this division: the adsorption energy of CO_2_ on Ce_2_O_4_-Co_3_O_4_(220) increases from −1.29 eV on perfect Co_3_O_4_(220) to −2.75 eV, and the adsorption energy of H_2_ on Ni_4_/Ce_2_O_4_-Co_3_O_4_(220) is also stronger than on Ni_4_-Co_3_O_4_(220) (−3.00 vs. −2.16 eV). Thus, CeO_2_ mainly enhances CO_2_ capture and activation, Ni promotes H_2_ dissociation and CH_4_ selectivity, and Co_3_O_4_ provides the basic oxide framework and CO_2_ adsorption sites; together, these explain the high conversion and CH_4_ yield of this composite oxide in low-temperature methanation.

CoO_x_/CeO_2_ differs from NiO/CeO_2_ in that its oxidation state and morphology exhibit significant dynamics under CO_2_/H_2_. Hu et al. constructed hollow Ni/CeO_2_-Co_3_O_4_ for CO_2_ methanation, emphasizing that the synergy of Co_3_O_4_ with Ni/CeO_2_ enhances CO_2_ adsorption and activation, improving methanation performance [139]. Deng et al., using in situ XAFS/XRD/DRIFTS and environmental TEM, visualized the size-dependent dynamic behavior of CoO_x_ nanoparticles supported on CeO_2−_δ(100) under CO_2_ hydrogenation: when the atmosphere switched from H_2_ to H_2_/CO_2_ (250 °C), small CoO_x_ nanoparticles (<2.5 nm) rapidly transformed from 3D pyramidal shapes to single-atomic-layer 2D structures attached to the CeO_2−_δ(100) surface, while larger particles (>3 nm) showed essentially no morphological change. This reversible 3D→2D transformation increases binding sites for CO_2_ or intermediates and alters methane selectivity [104].

#### 3.3.6. In_2_O_3_/CeO_2_: An Oxide–Oxide Methanol Pathway with Coexisting Activity and Deactivation

In_2_O_3_ is an important oxide catalyst for CO_2_-to-methanol, and its combination with CeO_2_ has both activity potential and water-stability issues. In Table 4, Ng et al. performed kinetic modeling of CO_2_ hydrogenation to methanol over In_2_O_3_/CeO_2_, using a dual-site Langmuir-Hinshelwood mechanism and MARI analysis on 13 steady-state datasets (200–300 °C, 1–50 bar, GHSV 5893–58,938 h^−1^), pointing out that H* mainly resides on H_2_ adsorption site S_1_, while formate (CHO_2_*) and CO_2_* mainly reside on CO_2_ site S_2_; formate was identified as the key surface intermediate [132]. Sharma et al., from a stability perspective, pointed out that In_13_/CeO_2_ is more prone to form In(OH)_3_ and OH accumulation in the presence of water, leading to deactivation, indicating that the methanol pathway over In_2_O_3_/CeO_2_ requires simultaneous optimization of oxygen vacancy activity and resistance to water-induced hydroxylation [136].

These results show that oxide–oxide interfaces do not necessarily require metal nanoparticles to catalyze methanol, but their active sites often depend on the dynamic balance among oxygen vacancies, hydroxyls, and water. The hydrophilicity and reducibility of CeO_2_ can promote CO_2_ activation, but may also promote OH accumulation and deactivation; therefore, regulation via ZrO_2_, MgO, or hydrophobic interfaces is needed to avoid water-induced structural degradation [131,132,136].

Within the different reaction classes summarized in Table 5, mixed-oxide/CeO_2_ catalysts provide several routes for modifying CO_2_ adsorption, redox properties, intermediate conversion, and water removal. CaO–Cu/CeO_2_ and CeO_2_-confined NiFe_2_O_4_ are mainly associated with CO production, whereas Ni–CeO_2_/γ-Al_2_O_3_ and Ni/CeO_2_–Co_3_O_4_ favour CH_4_ formation. CuO/CeO_2_-based composites and CZZC are primarily investigated for methanol synthesis. These entries demonstrate the range of functions that can be introduced through oxide–ceria combinations, but they do not establish a common activity order because the systems were evaluated under different reaction conditions and reported different performance metrics. Therefore, the table should be interpreted as a map of material functions and operating windows rather than as a direct performance ranking.

Compared with metal nanoparticles, oxide–oxide interfaces place greater emphasis on CO_2_ adsorption, surface hydrogen/oxygen migration, and water removal. From a broader perspective, composite oxide/CeO_2_ systems extend CeO_2_-based catalysts from conventional metal–support interfaces to multi-oxide functional partitioning. This framework helps explain why some systems have high initial activity but poor stability, and also helps distinguish the different contributions of adsorbents, redox promoters, and true hydrogenation sites.

### 3.4. Cross-Category Comparison of Selectivity-Controlling Site Ensembles

Across single-metal, bimetallic, and mixed-oxide catalysts, product selectivity can be interpreted as competition among three pathway branches: conversion of CO_2_-derived species through formate/methoxy intermediates, formation and desorption of CO through RWGS-related routes, and further hydrogenation or coupling of retained CO*. The same ceria defect can therefore promote different products when it is coupled to different metal ensembles. Cu–CeO_2_ interfaces can favour oxygenate chemistry when interfacial sites sustain formate-derived intermediate conversion and suppress RWGS, whereas Ni-containing ensembles more readily drive CO* hydrogenation toward CH_4_ [41,78].

Vacancy location and exposed facets regulate the first branch by determining whether CO_2_ is activated in a convertible form or trapped as strongly bound carbonate [34,43]. Metal nuclearity regulates the subsequent fate of CO*. Smaller Rh species suppress the Sabatier pathway and favour CO formation, whereas larger Rh particles and stronger hydrogen spillover increase CH_4_ formation [44]. Pd dimers and Ti-stabilized Rh single atoms provide a different nuclearity regime, in which proximate CO* and CH_x_* intermediates can be retained for C–C coupling and ethanol formation [92,95].

Interfacial structure is therefore more informative than the magnitude of metal-support interaction alone. Strong Cu–CeO_2_–ZrO_2_ interaction can enhance methanol synthesis when it creates accessible Cu-support sites and accelerates formate-to-methoxy conversion [140]. Conversely, excessively strong Pd–Cu/CeO_2_ interaction can reconstruct the alloy, increase competitive CO_2_/H_2_ adsorption, and lower methanol productivity [114]. Likewise, ZnO incorporation into Ni/CeO_2_ suppresses the methanation tendency and shifts the product distribution toward CO, showing that oxide modification can alter pathway branching without changing the primary metal [111].

This comparison also defines the limits of the current literature. Ex situ Ce^3+^ fractions, Raman defect bands, and adsorption capacities cannot independently identify the selectivity-controlling site. Mechanistic assignments should therefore be strengthened by correlating operando intermediate evolution with metal nuclearity, interfacial coordination, and product formation under identical reaction conditions. This approach distinguishes a reactive interfacial vacancy from a spectator defect or a site that merely accumulates carbonate species.

## 4. Conclusions and Future Perspectives

Across the reviewed systems, assignments based on species observed under operating conditions provide the most immediate basis for discussing reaction intermediates, whereas structure–performance trends and theoretical calculations mainly constrain the possible roles and energetic feasibility of proposed active motifs. Their integration is therefore essential for avoiding overinterpretation of any single descriptor, such as the Ce^3+^ fraction or the apparent oxygen-vacancy concentration.

Overall, CeO_2_-based catalysts should be regarded as dynamic interfaces that couple CO_2_ activation, hydrogen transfer, intermediate stabilization and product desorption. It is these structural features that allow CeO_2_ to simultaneously participate in multiple steps, CO_2_ adsorption polarization, oxygenate intermediate stabilization, lattice oxygen migration, H species spillover, and water desorption, on the same interface, thereby significantly altering the activity, selectivity, and stability of CO and CO_2_ hydrogenation reactions.

From the single-metal perspective, Cu/CeO_2_ and Ni/CeO_2_ represent two typical directions of CeO_2_ synergy. In contrast, Au, Pd, Rh, Ru, Co, Ir, and other metal/CeO_2_ systems display a broader selectivity window: Au and some Pd systems can favor CO or formate pathways; Pd dimers and Rh single atoms/clusters can direct ethanol and other C_2_ oxygenates, while Ru, Co, and Ir exhibit strong hydrogenation ability in methanation. This shows that CeO_2_ does not exert the same promotional effect on all metals, but reshapes the product outlet by tuning metal valence, nuclearity, interfacial oxygen vacancies, and local oxygen chemical potential.

Bimetallic/CeO_2_ systems further prove that “synergy” should not be simply understood as the sum of two metal activities, but as a functional division of labor among the main metal, the second metal, and CeO_2_ defect sites. Systems such as PdZn, CuZn, PdCu, CuNi, NiCo, NiFe, and single-atom Y or Ti-doped Rh demonstrate that the second metal can alter the d-band electronic structure, H_2_ dissociation ability, CO* adsorption strength, and C-O/C-C bond transformation pathways of the main metal; simultaneously, it also affects the Ce^3+^/Ce^4+^ ratio, oxygen vacancy concentration, and regenerability of oxygen vacancies on the CeO_2_ surface.

Composite oxide/CeO_2_ systems redistribute CO_2_ adsorption, H_2_ activation, redox cycling and water removal among multiple components. These results collectively indicate that the design goal of CeO_2_ composite oxides should not be merely increasing oxygen vacancy numbers, but achieving a reasonable balance among basic sites, redox-active vacancies, formate-stabilizing sites, metal/oxide interfaces, and H_2_O desorption capability.

More importantly, the different sections actually point to the same design principle: the defect chemistry of CeO_2_ only acquires catalytic significance when dynamically coupled with metal hydrogenation ability, oxide acidity/basicity, and reaction atmosphere. Isolated Ce^3+^ fraction, oxygen vacancy concentration, CO_2_-TPD intensity, or metal dispersion are insufficient to predict final selectivity; comparable descriptors should simultaneously include site proximity, stability under reaction conditions, and intermediate turnover capacity.

Despite substantial progress, several key challenges remain in CeO_2_-assisted CO/CO_2_ hydrogenation. First, the true active sites are often dynamically generated under reaction conditions, rather than the static structures observed in catalyst characterization. H_2_ reduction, CO_2_/H_2_ co-feeding, water formation, CO coverage, and strong metal-support interaction can all cause metal particle reconstruction, single-atom migration, CeO_2−x_ overlayer formation, or oxygen vacancy redistribution. Therefore, future research should rely more on operando/near-ambient-pressure XPS, in situ XAFS, in situ DRIFTS, environmental TEM, isotopic transient kinetics, and steady-state isotopic exchange to directly track the simultaneous evolution of Ce^3+^/Ce^4+^, oxygen vacancies, metal valence, and key intermediates during reaction. Recent advances in two-dimensional materials also offer a possible route towards more informative operando measurements. The demonstration of a dual-mode sensor based on two-dimensional InSe illustrates the potential of low-dimensional semiconductors to integrate complementary sensing signals [147]. For CO/CO_2_ hydrogenation, this concept may inspire reactor-compatible platforms that correlate fluctuations in CO_2_, CO, H_2_O, or H_2_ with reaction-induced electronic changes at catalyst interfaces. However, two-dimensional materials have not yet been established as direct operando probes or universally beneficial catalyst components for CeO_2_-based hydrogenation. Their value should therefore be assessed through stability, selectivity, and spectroscopic correlation under realistic reaction conditions.

Second, the design of CeO_2_ catalysts needs to move from the qualitative description of “more oxygen vacancies are better” to quantifiable descriptors. Different oxygen vacancies play different roles in the reaction: some promote CO_2_ adsorption, some participate in MvK-type RWGS, some stabilize formate/methoxy intermediates, and others convert to OH species in the presence of water and cause deactivation. Future studies should distinguish vacancy concentration from vacancy reactivity, formation energy, mobility, redox activity and interfacial location, and correlate these parameters with TOF, apparent activation energy, product selectivity, and long-term stability. Combining DFT, microkinetic modeling, and machine learning screening holds promise for establishing a predictable design map for CeO_2_-based catalysts.

Third, selectivity control remains the core issue for moving CeO_2_ synergistic systems toward high-level applications. For methanol synthesis, a balance must be struck among CO_2_ activation, H_2_ dissociation, formate/methoxy hydrogenation, and RWGS suppression; for methanation, low-temperature H_2_ activation and CO* deep hydrogenation need to be enhanced while avoiding sintering or excessive encapsulation of Ni, Ru, Co, and other metals; for CO and C_2_ oxygenate production, excessive hydrogenation must be suppressed and spatial sites suitable for CO* retention, CH_x_* generation, and C-C coupling must be constructed. Future directions worthy of emphasis include atomic-scale bimetallic sites, single-atom-oxygen vacancy Lewis acid-base pairs, metal dimers, confined alloys, and tandem/spatially partitioned catalysts, to achieve programmable selectivity conversion from C_1_ products to methanol, ethanol, and other high-value oxygenates.

Fourth, stability and adaptability to realistic conditions should become key evaluation criteria in subsequent studies. Many CeO_2_-based catalysts perform excellently under ideal CO_2_/H_2_ conditions, but may suffer from metal sintering, carbon deposition, hydroxyl coverage, oxygen vacancy depletion, support phase transformation, and excessive SMSI encapsulation under high conversion, water vapor, CO coexistence, impurities, thermal cycling, or long-term operation. Therefore, future reports should not only provide short-term activity and selectivity, but also systematically compare at least tens to hundreds of hours of stability, cyclic regeneration capability, resistance to water/impurities, and structural evolution after deactivation. For integrated CO_2_ capture-conversion systems, capture capacity, release kinetics, catalytic conversion efficiency, and cyclic mechanical stability should be evaluated simultaneously, avoiding the simplistic equation of adsorbent performance with catalytic performance.

Finally, from an application perspective, the future development of CeO_2_ synergistic catalytic systems should address the practical needs of low-carbon chemical synthesis and distributed CO_2_ utilization. Low temperature, low pressure, high space velocity, low H_2_/CO_2_ ratio, CO-containing or dilute CO_2_ feeds, and fluctuating renewable H_2_ conditions will more closely resemble real industrial or energy-system scenarios. To this end, powder catalyst research should be advanced to shaped catalysts, structured reactors, membrane reactors, and integrated capture-conversion processes, combined with techno-economic analysis and life-cycle assessment to judge the actual carbon-reduction potential. Overall, the advantage of CeO_2_ lies not just in providing oxygen vacancies, but in its ability to couple metal active sites, oxide defect sites, and reaction atmosphere into a dynamically tunable interface. Only by elevating this dynamic interface from empirical observation to quantifiable design can CeO_2_-based catalysts play a greater role in research and applications for CO/CO_2_ hydrogenation to methanol, methane, CO, and C_2_^+^ oxygenates.

## Figures and Tables

**Table 1:** Scope and distinguishing features of representative reviews relevant to CeO_2_-based CO and CO_2_ hydrogenation.

No.	Representative Review	Primary Focus	Scope Relative to This Review	Distinct Contribution of the Present Review	Ref.
1	Montini et al. (2016) Chemical Reviews	Foundational chemistry of CeO_2_: fluorite structure, Ce^4+^/Ce^3+^ redox, oxygen storage/release, oxygen vacancies, and broad catalytic applications.	A broad CeO_2_ materials review; it does not develop a CO/CO_2_ hydrogenation-centered framework or compare product selectivity across metals.	Links CeO_2_ defect chemistry directly to CO/CO_2_ hydrogenation steps, including CO_2_ activation, intermediate stabilization, H_2_ transfer, water removal, and selectivity–stability trade-offs.	[16]
2	Trovarelli & Llorca (2017) ACS Catalysis	Nanoscale CeO_2_: particle size, morphology, exposed facets, surface reconstruction, oxygen reactivity, and metal–support interactions.	Emphasizes morphology–activity relationships, but does not systematically compare Cu, Ni, Co, Ru, Pd, and Rh in CO/CO_2_ hydrogenation.	Establishes a facet–defect-location–metal nuclearity/interface–product-selectivity relationship and emphasizes dynamic reconstruction under reaction atmospheres.	[28]
3	Yan et al. (2022) Chem Catalysis	CeO_2_-supported noble-metal single atoms, nanoclusters, and nanoparticles: synthesis, structure, metal–support interactions, and catalytic properties.	Focused on noble metals and diverse reactions (e.g., CO oxidation, WGS, hydroformylation); it is not a comprehensive CO/CO_2_ hydrogenation review.	Places noble metals alongside Cu/Ni/Co systems and compares how metal identity, nuclearity, interfacial structure, and CeO_2_ defects direct CO, CH_4_, CH_3_OH, and C_2_ oxygenate formation.	[29]
4	Tan et al. (2023) Chemical Engineering Journal	Bifunctional CO/CO_2_ hydrogenation catalysts: component integration, intercomponent distance, migrating/non-migrating species, and C_2_^+^ products.	Provides a general bifunctional-catalyst design framework; CeO_2_ defects, defect-probe limitations, and long-term operating stability are not the central themes.	Translates bifunctional design into CeO_2_ defect–metal interfaces, while integrating defect characterization, reaction pathways, selectivity control, and realistic operating constraints.	[27]
5	Liu et al. (2025) Carbon Neutralization	Ni/CeO_2_ for CO_2_ methanation: low-temperature activity, Ni–CeO_2_ interactions, vacancy engineering, coking/sintering, and industrial challenges.	Restricted mainly to Ni/CeO_2_ and CH_4_ synthesis; it does not cover the broader metal, product, and CO-hydrogenation space.	Extends the discussion from Ni/methanation to multiple metals, products, and CO/CO_2_ hydrogenation, using a unified defect-location–dynamic-interface–application framework.	[30]
Present review	CO and CO_2_ Hydrogenation over CeO_2_ and CeO_2_-Based Composite Catalysts (Nanomaterials, revised manuscript)	Function-oriented coverage of pristine CeO_2_, single-metal/CeO_2_, bimetallic/CeO_2_, and CeO_2_–metal–oxide composites.	—	Four main advances: (i) simultaneous CO and CO_2_ hydrogenation coverage; (ii) defect location, type, reactivity, and dynamic evolution rather than vacancy concentration alone; (iii) explicit comparison of XPS, Raman, EPR, TPR/TPD, and operando probes; and (iv) coupling of descriptors to water/CO tolerance, impurities, sintering, regeneration, structured reactors, and TEA/LCA.	

**Table 4:** Overview of reaction conditions and results of catalytic tests at steady state over In_2_O_3_/CeO_2_ catalyst for 13 experimental runs. Experiment 1–5 (Temperature variation), A–D (Pressure variation), S_1_–S_4_ (Space velocity variation).

Exp	Temperature (°C)	Pressure (bar)	^1^ GHSV (hr^−1^)	^2^ r_moh_ (mol/s/kg.cat)	^3^ Xc_o2_ (%)	^4^ S_moh_ (%)	^5^ Sc_o_ (%)
1	200	50	17,681	5.72 × 10^−10^	29.50%	19.41%	73.04%
2	225	50	17,681	1.06 × 10^−9^	67.99%	14.47%	74.74%
3	250	50	17,681	3.24 × 10^−9^	77.31%	7.03%	82.94%
4	275	50	17,681	5.21 × 10^−9^	46.60%	4.90%	86.00%
5	300	50	17,681	1.13 × 10^−8^	32.90%	94.34%	0.00%
A	300	1	17,681	4.58 × 10^−11^	68.07%	68.23%	0.00%
B	300	20	17,681	3.56 × 10^−10^	67.86%	49.34%	0.00%
C	300	40	17,681	9.08 × 10^−10^	31.24%	35.78%	0.00%
D	300	50	17,681	2.02 × 10^−9^	32.90%	94.34%	0.00%
S1	300	50	5893	5.72 × 10^−10^	17.55%	11.74%	80.44%
S2	300	50	11,788	1.06 × 10^−9^	17.48%	24.74%	82.24%
S3	300	50	29,469	5.21 × 10^−9^	28.71%	96.26%	0.00%
S4	300	50	58,938	1.13 × 10^−8^	34.65%	98.51%	0.00%

**Note:** Table 4 is reproduced from Ref. [132], with permission from Elsevier, and retains the original units and normalization basis reported in that study. Pressure is expressed in bar, GHSV in h^−1^, and the methanol formation rate, r_meoh_, in mol s^−1^ kg_cat^−1^. XCO_2_, SMeOH, and SCO denote CO_2_ conversion, methanol selectivity, and CO selectivity, respectively. These source-specific parametric data are presented as reported in the original study and should not be directly compared with the catalyst-performance data summarized in Table 2, Table 3 and Table 4. ^1^—Gas hourly space velocity; ^2^—Synthesis rate of methanol; ^3^—Conversion of CO_2_; ^4^—Selectivity of methanol; ^5^—Selectivity of CO.

**Table 5:** Reported catalytic performance of metal–oxide/CeO_2_ composite catalysts, organized by target reaction.

Catalyst	Product	Temperature (°C)	Pressure (MPa)	GHSV/Space Velocity	Conversion (%)	Selectivity (%)	TOF/STY (Type and Units)	Stability Test Duration (h)	Ref.
CaO-Cu/CeO_2_-R	CO	650 °C	~0.1	-	75 (CO_2_)	100 (CO)	Yield: 9.72 mmol·g^−1^	~4.6 h	[142]
20CeO_2_-NiFe_2_O_4_	CO	700 °C	0.1	-	80.85 (CO_2_)	~100(CO)	-	50 h	[118]
Ni-3CeO_2_/γ-Al_2_O_3_	CH_4_	300 °C	0.1	3000 mL·gcat^−1^·h^−1^	95.0 (CO_2_)	>99.5 (CH_4_)	-	150 h	[75]
Ni/CeO_2_-Co_3_O_4_	CH_4_	300 °C	~0.1	-	85.4 (CO_2_)	>80 (CH_4_)	-	60 h	[139]
CuCe/S1	CH_3_OH	240 °C	3.0	8000 mL·gcat^−1^·h^−1^	-	58(CH_3_OH)	STY: 2.72mmol_MeOH·g_Cu^−1^·h^−1^	100 h	[40]
Cu(5)/ZnO-CeO_2_(0.05)	CH_3_OH	250 °C	3.0	60,000 mL·gcat^−1^·h^−1^	~8 (CO_2_)	75 (CH_3_OH)	-	-	[134]
CuO/CeO_2_-H	CH_3_OH	260 °C	3.0	10,000mL·gcat^−1^·h^−1^	15.6 (CO_2_)	92.4 (CH_3_OH)	-	-	[60]
CZZC (Cu-ZnO-ZrO_2_-CeO_2_)	CH_3_OH	250 °C	4.5	5700 mL·gcat^−1^·h^−1^	28.7 (CO_2_)	44.3 (CH_3_OH)	-	-	[129]

**Note:** Values are compiled from the original studies with unit harmonization where unambiguous. Temperature is given in °C and pressure in MPa. STY values were converted to mmol of product per gram of the reported catalyst or metal basis per hour when the molecular weight and normalization basis were available. Yield values without a time basis were retained in their original form. A dash (-) indicates that the corresponding value was not reported. The entries are intended to show reported operating windows and product tendencies rather than establish a direct performance ranking.

## Data Availability

No new data were created or analyzed in this study. Data sharing is not applicable to this article.
